# Emergency Department Programs to Support Medication Safety in Older Adults: A Systematic Review and Meta-Analysis

**DOI:** 10.1001/jamanetworkopen.2025.0814

**Published:** 2025-03-11

**Authors:** Rachel M. Skains, Jane M. Hayes, Katherine Selman, Yue Zhang, Phraewa Thatphet, Kazuki Toda, Bryan D. Hayes, Carla Tayes, Martin F. Casey, Elizabeth Moreton, Richard E. Kennedy, Sangil Lee, Shan W. Liu

**Affiliations:** 1 Department of Emergency Medicine, Heersink School of Medicine, The University of Alabama at Birmingham; 2 Geriatric Research, Education, and Clinical Center, Birmingham VA Medical Center, Birmingham, Alabama; 3 Department of Emergency Medicine, Massachusetts General Hospital, Harvard Medical School, Boston; 4 Department of Emergency Medicine, Cooper University Hospital, Cooper Medical School at Rowan University, Camden, New Jersey; 5 Division of Gerontology, Geriatrics, and Palliative Care, Department of Medicine, The University of Alabama at Birmingham; 6 Department of Emergency Medicine, Faculty of Medicine, Khon Kaen University, Khon Kaen, Thailand; 7 Department of Emergency Medicine/Internal Medicine, Christiana Care Health System, Newark, Delaware; 8 Department of Emergency Medicine, UNC School of Medicine, The University of North Carolina at Chapel Hill; 9 Department of Emergency Medicine, Carver College of Medicine, The University of Iowa, Iowa City

## Abstract

**Importance:**

Given that older adults are at high risk for adverse drug events (ADEs), many geriatric medication programs have aimed to optimize safe ordering, prescribing, and deprescribing practices.

**Objective:**

To identify emergency department (ED)–based geriatric medication programs that are associated with reductions in potentially inappropriate medications (PIMs) and ADEs.

**Data Sources:**

A systematic search of Scopus, Embase, PubMed, PsycInfo, ProQuest Central, CINAHL, AgeLine, and Cochrane Library was conducted on February 14, 2024, with no date limits applied.

**Study Selection:**

Randomized clinical trials or observational studies focused on ED-based geriatric (aged ≥65 years) medication programs that provide ED clinician support to avoid PIMs and reduce ADEs.

**Data Extraction and Synthesis:**

Preferred Reporting Items for Systematic Reviews and Meta-Analyses guidelines for abstracting data and the Cochrane risk-of-bias tool were used to assess data quality and validity. Abstract screening and full-text review were independently conducted by 2 reviewers, with a third reviewer acting as an adjudicator.

**Main Outcomes and Measures:**

Process (ordering, prescribing, and deprescribing PIM rates) and clinical (ADE, health care utilization, and falls) outcomes.

**Results:**

The search strategy identified 3665 unique studies, 98 were assessed for eligibility in full-text review, and 25 studies, with 44 640 participants, were included: 9 clinical pharmacist reviews (with 28 360 participants), 1 geriatrician teleconsultation (with 50 participants), 8 clinician educational interventions (with 5888 participants), 4 computerized clinical decision support systems (CDSS; with 9462 participants), and 3 fall risk–increasing drug (FRID) reviews (with 880 participants). Clinical pharmacist review was not associated with decreased hospital admission or length of stay, but 2 studies showed a 32% reduction in PIMs from deprescribing (odds ratio [OR], 0.68 [95% CI, 0.50-0.92]; *P* = .01). One study also found that ED geriatrician teleconsultation was associated with enhanced deprescribing of PIMs. Three clinician educational intervention studies showed a 19% reduction in PIM prescribing (OR, 0.81 [95% CI, 0.68-0.96]; *P* = .02). Two computerized CDSS studies showed a 40% reduction in PIM ordering (OR, 0.60 [95% CI, 0.48-0.74]; *P* < .001). FRID reviews were not associated with reduced time to first fall or fall recurrence at 12 months.

**Conclusions and Relevance:**

In this systematic review and meta-analysis of ED-based geriatric medication safety programs, a multidisciplinary team, including clinical pharmacists and/or geriatricians, was associated with improved PIM deprescribing. Furthermore, computerized CDSS, alone or in combination with ED clinician education, was associated with enhanced geriatric ordering and prescribing practices. These findings will inform the Geriatric ED Guidelines version 2.0 update.

## Key Points

**Question:**

Are geriatric medication programs based in the emergency department (ED) associated with reduced potentially inappropriate medications (PIMs) and adverse events for adults aged 65 years or older?

**Findings:**

This systematic review and meta-analysis of 25 eligible studies with 44 640 participants found that multidisciplinary approaches, including clinical pharmacists and geriatricians, were associated with improved PIM deprescribing among older adults but not with hospital outcomes, while computerized clinical decision support systems, with or without ED clinician education, were associated with enhanced geriatric ordering and prescribing practices by reducing PIMs. However, medication reviews targeting fall risk–increasing drugs were not associated with reduced falls in older adults.

**Meaning:**

These findings will inform the implementation of ED-based geriatric medication safety programs in updating the Geriatric ED Guidelines version 2.0.

## Teaser

This systematic review and meta-analysis evaluates whether any emergency department–based medication programs for adults aged 65 years and older are associated with lower adverse process or clinical outcomes.

## Introduction

Adults aged 65 years and older account for 26.8 million (20.4%) annual emergency department (ED) visits.^1^ Older adults are susceptible to high-risk medications in the ED setting due to geriatric syndromes requiring complex medical decision-making.^2,3^ Due to polypharmacy (concomitant use of ≥5 medications), comorbidities, and physiologic changes of aging, older adults are predisposed to adverse drug events (ADEs), which are associated with ED revisits, hospitalization, and mortality.^4,5,6,7,8,9^ Concerningly, rates of inappropriate prescription drug use and polypharmacy are rising among older adults.^10,11^ Almost half of older patients are discharged from the ED with at least one new prescription medication, and more than 85% of adults aged 60 years and older report using prescription drugs in the past 30 days.^12,13,14^ Concerns over safe medication use has led to explicit criteria for potentially inappropriate medications (PIMs) in older adults, such as the American Geriatrics Society (AGS) Beers Criteria and Screening Tool of Older Person’s Prescriptions/Screening Tool to Alert Doctors to Right Treatment (STOPP/START) criteria.^15,16,17,18^ In recent meta-analyses, older adults were 91%, 60%, and 26% more likely to have ADE-related hospitalization, functional decline, and ADE, respectively, when prescribed a PIM, and this risk increased with increasing number of PIMs.^6,7^

Safer medication use and management has been a research priority for high-quality geriatric emergency care for nearly 20 years.^19,20,21^ More recently, the Centers for Medicare & Medicaid Services identified responsible medication management monitoring PIM use in older adults as a core domain of age-friendly hospitals.^22^ In 2014, American College of Emergency Physicians (ACEP), Emergency Nurses Association, Society for Academic Emergency Medicine, and AGS-endorsed geriatric ED guidelines outlining policies and protocols for medication management, including screening for polypharmacy and high-risk medication use with established medication reconciliation tools and engaging a multidisciplinary team, including pharmacists and geriatric specialists.^23^ Santangelo et al^24^ reported that 69% of level I and II accredited geriatric EDs, which meet certain quality-of-care criteria by ACEP’s geriatric ED accreditation program, had care processes to minimize the use of PIMs and 60% had medication reconciliation protocols leveraging pharmacists.^24^ Although several studies have evaluated the impact of geriatric medication program interventions to optimize safe medication practices for older adults being treated in the ED, it is unknown how EDs can best facilitate responsible medication management to reduce PIMs and associated ADEs in older adults.^25,26^ The Geriatric ED Guidelines 2.0 is a multidisciplinary initiative to update the 2014 iteration. As part of this effort, we systematically reviewed the published literature to identify which ED-based geriatric medication programs were associated with reduced PIMs and ADEs.

## Methods

This systematic review and meta-analysis is reported in accordance with the Preferred Reporting Items for Systematic Review and Meta-Analyses (PRISMA) reporting guidelines.^27^ It was not registered; a protocol was not published.

### Search Strategy

We conducted literature searches using strategies created by a medical librarian (E.M.) to identify studies that analyzed the impact of ED-based geriatric medication programs providing support for ED clinicians to avoid PIMs. The search strategies (eMethods in Supplement 1) were established using a combination of keywords.

### Inclusion and Exclusion Criteria

We included studies of ED medication programs targeting patients aged 65 years and older. We defined geriatric medication programs broadly as clinical pharmacist review, clinician educational interventions, geriatrician teleconsultations, computerized clinical decision support systems (CDSS), or high-risk medication reviews that aid ED clinicians when ordering, prescribing, and/or deprescribing medications during the ED stay or at ED discharge.

We excluded studies that were not randomized clinical trials (RCTs), nonrandomized interventional studies, or observational cohort studies, including case series and reports, systematic and scoping reviews, abstracts, and dissertations and theses. We excluded studies if the intervention was not initiated in the ED, did not provide ED clinician support to avoid PIMs, or lacked a comparison group. The initial search was run August 8, 2022, and updated February 14, 2024, with no date limits applied. We used Scopus, Embase, PubMed, PsycInfo, ProQuest Central, CINAHL, AgeLine, and Cochrane Library.

### Study Selection

Duplicate studies were first automatically identified by the citation manager EndNote version X8 (Clarivate) by comparing title, author, and year for exact matches. After the first pass of exclusions, all remaining studies were then reviewed by the librarian (E.M.) for similarities in title, author, and year, then by abstract, to identify and remove any duplicates not found by EndNote due to differences in formatting. Unique citations were then exported to Covidence, a systematic review software, which also checked for duplicates upon import in the title, year, author, and volume fields. Two team members (of B.D.H., K.S., S.L., K.T., P.T., J.M.H., C.T., M.F.C., R.M.S., and S.W.L.) independently screened reference titles and abstracts and reviewed the full texts of studies to determine final inclusion. Any disagreements were adjudicated by a third reviewer (another of the authors listed previously).

### Data Extraction and Quality Assessment

Qualifying studies not published in English were translated. Four team members independently extracted data from included studies (B.D.H., K.T., S.W.L., and R.M.S.). Outcomes included ordering, prescribing, and/or deprescribing rates, comparison of preintervention and postintervention PIM rates, and adverse event rates. Adverse events included but were not limited to ADEs, hospitalization, length of stay (LOS), mortality, ED revisit, delirium, and falls (eFigure 3 in Supplement 1).

The risk of bias (ROB) of each study was assessed using the Revised Cochrane Risk of Bias Tool for Randomized Trials (RoB 2.0) for RCTs and the Risk of Bias in Non-Randomized Studies of Interventions (ROBINS-I) for observational studies.^28,29^ Four team members (J.M.H., S.L., K.S., and P.T.) participated in ROB analyses. For both data extraction and ROB assessment, each study was analyzed independently by 2 team members and disagreements were resolved by discussion.

### Statistical Analysis

For each study, odds ratios (ORs) and hazard ratios (HRs) with 95% CIs were extracted. If necessary, ORs were calculated based on sample numbers reported in the study results. For studies reporting hospital LOS as median and IQR, the mean and SD were estimated using a previously reported method.^30^

A random-effects meta-analysis model was utilized to better account for heterogeneity between studies.^31^ Heterogeneity between studies was assessed using Cochran *Q*.^32^ Weighting by sample size was used to avoid excessive influence of smaller studies. Funnel plots and Fail-Safe N were used to assess presence of study bias and robustness of results (eFigure 1 in Supplement 1).^33,34^ All analyses were conducted using the metafor package^35^ in R Studio version 2024.12.0+467 (R Project for Statistical Computing). Statistical significance was set at α = .05, and all tests were 2-tailed.

## Results

### Included Studies and ROB

**Figure 1.**
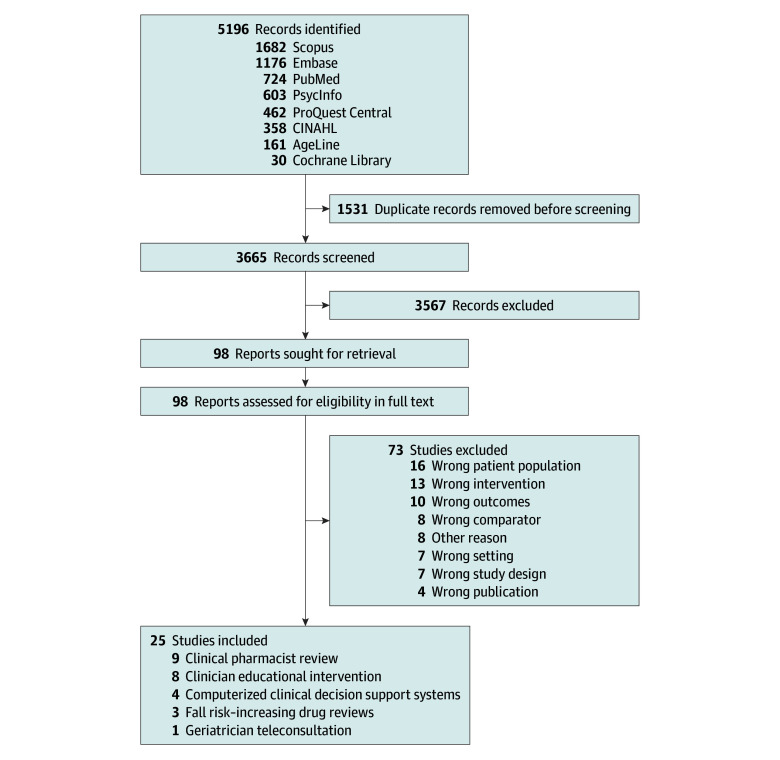
Flowchart of Included Studies

**Figure 2.**
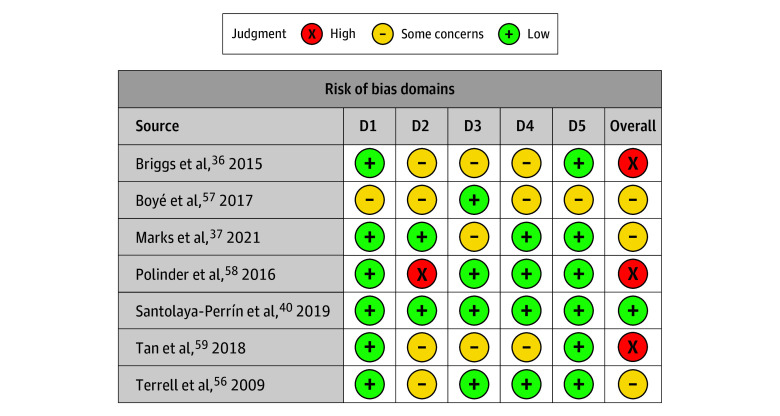
Revised Cochrane Risk of Bias Tool for Randomized Trials for Randomized Clinical Trials Figure was created using robvis,^60^ a web app designed for visualizing risk-of-bias assessments performed as part of a systematic review. Domain (D) 1 indicates bias arising from the randomization process; D2, bias due to deviations from intended intervention; D3, bias due to missing outcome data; D4, bias in measurement of the outcome; and D5, bias in selection of the reported result.

**Figure 3.**
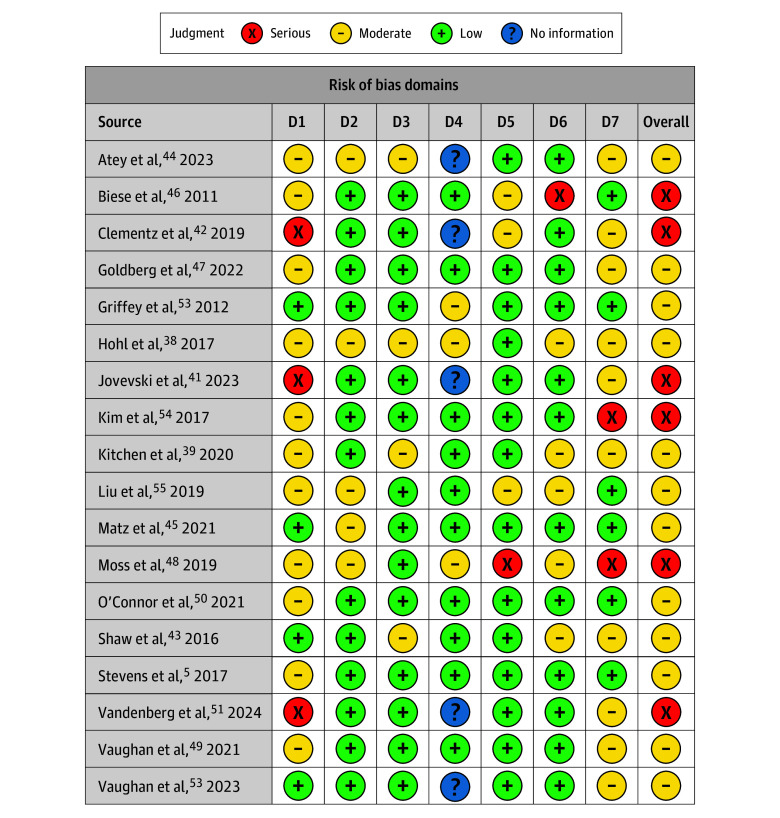
Risk of Bias in Nonrandomized Studies of Interventions or Observational Studies. Figure was created using robvis,^60^ a web app designed for visualizing risk-of-bias assessments performed as part of a systematic review. Domain (D) 1 indicates bias due to confounding; D2, bias due to selection of participants; D3, bias in classification of interventions; D4, bias due to deviations from intended interventions; D5, bias due to missing data; D6, bias in measurement of outcomes; and D7, bias in selection of the reported result.

The literature search identified 5196 abstracts. After deduplication, 3665 unique abstracts remained. Of these, 3567 were excluded based on title and abstract review. We assessed the full text of 98 studies for eligibility. Ultimately, we included 25 studies (Figure 1)^5,36,37,38,39,40,41,42,43,44,45,46,47,48,49,50,51,52,53,54,55,56,57,58,59^ with 44 640 participants, published between 2009 and 2024. Included studies evaluated the following interventions: 9 clinical pharmacist review (with 28 360 participants),^36,37,38,39,40,41,42,43,44^ 1 geriatrician teleconsultation (with 50 participants),^45^ 8 clinician educational interventions (with 5888 participants),^46,47,48,49,50,51,52^ 4 computerized CDSS studies (with 9462 participants),^53,54,55,56^ and 3 fall risk–increasing drug (FRID) reviews (with 880 participants)^57,58,59^ (Table and eTable in Supplement 1). We determined that 1 study^40^ (4%) had low ROB, 15 studies^5,37,38,39,43,44,45,47,49,50,52,53,55,56,57^ (60%) had moderate or some concerns of ROB, and 9 studies^36,41,42,46,48,51,54,58,59^ (36%) had serious or high ROB (Figures 2 and 3).^60^

**Table.** Characteristics of Included Studies

Source (location)	Time frame	Patients, No.	Age, y	% Male	Study design	Intervention	Control	Primary outcome	Secondary outcomes	
**Clinical pharmacist review studies**										
Atey et al,^44^ 2023 (Australia)	June 2020 to May 2021	321	PPMC arm: median, 82.3 y; early BPMH arm: 80.1 y; usual care arm: 75.4 y	47%	Pragmatic concurrent controlled study	PPMC arm, redesigned process; early BPMH arm, modified process	Usual care arm, traditional standard of care	Fewer patients in the PPMC group (41%) were prescribed ≥1 PIM, despite being prescribed more drugs, than those in the early BPMH group (48%) and the usual care group (51%) upon ED departure (*P* = .04). The risk of using ≥1 PIM on ED departure decreased by 14.6% (95% CI, 12.4% to 17.8%) and 19.6% (95% CI, 16.8% to 23.7%) with PPMC when compared with the early BPMH group and the usual care group, respectively.	On ED departure, the median number of PIMs per patient (*P* = .05) and the median number of PIMs per medication (*P* = .04) were both significantly lower for the PPMC group than for the comparison groups. Neither the median number of PIM per patient nor the median number of PIM per prescribed medication changed significantly within the comparison group on hospital discharge vs at baseline.	
Briggs et al,^36^ 2015 (Australia)	October 2011 to June 2012	1021	Mean, 81 y	Not reported	Stratified RCT	Medication review by clinical pharmacist	Usual care	Odds of admission was lower in intervention group (OR, 0.68 [95% CI, 0.53 to 0.87]; *P* = .002). No evidence that intervention affected hospital LOS for admitted patients (difference in days, 0.09 [95% CI, 0.08 to 0.25]; *P* = .31), rate of re-presentation (difference, 0.08% [95% CI, 0.12% to 0.28%]; *P* = .44), or admission to an aged care facility. GPs adopted 49% of pharmacists’ recommendations.	Admission rate was 9 pp lower in the intervention group compared with the control group (53% vs 62%; *P* = .003).	
Clementz et al,^42^ 2019 (France)	February to October 2018	252	≥75 y	Not reported	Longitudinal and comparative study	Early in-hospital clinical pharmacist-led medication review and reconciliation (clinical pharmacy services)	Patients admitted to OAEM unit during the same period but outside the operating hours of the clinical pharmacist	Rate of unplanned rehospitalizations within 90 d were lower in intervention group than control group (OR, 0.45 [95% CI, 0.26 to 0.79]; *P* = .005).	Unplanned rehospitalization rate within 30 d: aOR, 0.45 (95% CI, 0.26 to 0.95); *P* = .04; unplanned rehospitalization rate within 72 h: aOR, 0.24 (95% CI, 0.06 to 0.94); *P* = .04.	
Hohl et al,^38^ 2017 (Canada)	September 2011 to February 2012	10 807	Mean, 70 y	44%	Quasi-randomized design	Early in-hospital pharmacist-led medication review (obtaining medication history, discussing goals of therapy with the patient or caregiver, and reviewing medications to identify and resolve medication-related problems)	Usual care (nurse or physician-led medication reconciliation using electronic forms prepopulated with outpatient medication dispensing record)	Median hospital days was numerically lower (−0.48 days [95% CI, −0.96 to 0.00 days]; *P* = .06) in medication review group compared with usual care.	No significant association with ED revisits, admissions, readmissions, or mortality.	
Jovevski et al,^41^ 2023 (Indiana, United States)	October 2019 to February 2022	298	Mean, 82.4 y	98%	Retrospective before-and-after intervention pilot study	EHR-automated protocol for pharmacists to perform a medication reconciliation	Preintervention	The case rate of PIM deprescribing in the preintervention group was 11.1% compared with the case rate of PIM deprescribing in the postintervention group of 57.1% (*P* < .001).	Preintervention, 91% of PIMs remained unchanged at 60 days compared with only 49% postintervention (*P* < .05). Regardless of PIM identification, the 30-d primary care follow-up number increased postintervention: 31.5% vs 55.7% (*P* < .001). There was no change in 7- or 30-day subsequent ED visit or hospitalization rates. Mortality remained unchanged at 60 days.	
Kitchen et al,^39^ 2020 (Canada)	November 2011 to January 2013	10 783	Median, 70 y	44%	Population-based evaluation of a continuous QI project	Pharmacists completed a medication review	Physicians or nurses completed medication reconciliation	ED-based pharmacist-led medication review did not result in a significant change in total outpatient health services utilization. At 12 months, there was no change in the level or trend of total physician visits per 1000 patients between groups (*P* = .46 and *P* = .89, respectively).	No differences in the secondary outcomes of PCP visits or ED visits relative to standard of care in the 12 months after intervention.	
Marks et al,^37^ 2021 (United States)	NA	110	Median, 83 y	33%	Prospective RCT	Pharmacist-led motivational interviewing–based intervention (GAPcare): 20 min medication management session performed by pharmacists	Usual care: brochures on home safety	Total pharmacist recommendations, 219; partial or full uptake of recommendations: advisory, 47 (81% [95% Cl: 67% to 91%]); increased precaution, 22 (82% [95% CI: 60% to 95%]); specific actions, 83 (64% [95% CI, 53% to 74%]); decrease dose, 9 (44% [95% CI, 14% to 79%]); stop medication, 14 (64% [95% CI, 35% to 87%]);and use safer alternative, 23 (78% [95% CI, 47% to 83%]).	GAPcare: lower repeat fall-related ED visits (aIRR, 0.34 [95% CI, 0.15 to 0.76]) and all-cause ED visits (aIRR, 0.47 [95% CI, 0.29 to 0.74]).	
Santolaya-Perrín et al,^40^ 2019 (Spain)	October 2014 to June 2015	665	Mean, 78 y	47%	Multicenter RCT	Pharmacist reviewed chronic medications and identified PIMs based on the STOPP/START criteria	Usual care: chronic medication recorded, but identification of PIMs based on the STOPP/ START criteria was not performed	ARR of emergency visits and hospital admissions was 0.81 (95% CI, 0.62 to 1.06) at 3 months, 0.89 (95% CI, 0.70 to 1.13) at 6 months, and 0.95 (95% CI, 0.77 to 1.18) at 12 months.	NA	
Shaw et al,^43^ 2016 (Colorado, United States)	November 2012 to May 2013	4103	Mean, 77 y	42%	Retrospective cohort	CPS in the ED for seniors (EMBRACE)	Non-EMBRACE ED	CPS EMBRACE group (adjusted percentage, 19.6%) more likely to have 30-day ED return visit than control (adjusted percentage, 18.6%) (adjusted difference, 1.0 pp [95% CI, 3.2 to 5.9 pp]; aOR, 1.34 [95% CI, 1.02 to 1.76]).	Of the CPS EMBRACE group, 154 (45.0%) had at least 1 medication-related problem. No differences across groups in 90-day ED return visits, 90-day mortality, or 90-day health care expenditures.	
**Geriatrician teleconsultation study**										
Matz et al,^45^ 2021 (Germany)	November 2017 to February 2018	50	Mean, 82 y	60%	Prospective pilot study	Drug recommendations by telemedical consultation with geriatrician	Drug recommendations by ED physicians	Higher frequency of recommendations regarding changes to preexisting medications via geriatric telemedicine vs standard ED treatment (*P* < .001). Geriatricians intervened more often than ED physicians: discontinuation of a drug (*P* < .001); start of a new drug (*P* = .004); dose change of a drug (*P* = .001). Recommendations for immediate drug therapy were made more frequently by ED physicians (*P* = .04). Amount of medication taken by the patient was reduced compared with standard ED treatment (ED assessment *t*_49_ = 0.622 vs geriatrician’s assessment *t*_49_ = 4.165; *P* < .001). Geriatricians changed 53.9% of drugs (35/65) whereas ED physicians changed 12.3% (8/65).	The number of PIMs was lower compared with standard medical treatment (*P* < .001).	
**Clinician educational intervention studies**										
Biese et al,^46^ 2011 (North Carolina, United States)	2008 to 2010	No. depends on outcome	Mean, ≥65 y	Not reported	Prospective cohort pre-post intervention	Geriatric curriculum for EM residents	Before curriculum implementation	No change in percentage of older patients receiving chemical sedation before and after curriculum (5.4% vs 4.5%; *P* = .47).	Before and after curriculum total urinary Foley catheter use, 7.4% vs 5.9%, *P* = .30. Total inappropriate urinary Foley catheter use: 16.3% vs 2.1%, *P* = .03.	
Goldberg et al,^47^ 2022 (Rhode Island, United States)	July 2018 to January 2021	No. not reported	Mean, ≥65 y	Not reported	Quasi-experimental interrupted time series	EQUIPPED medication safety program implementation	Preimplementation of EQUIPPED	PIMs at the preimplementation compared with postimplementation time periods had a rate 1.11 times greater (95% CI, 1.03 to 1.21; *P* < .01). In the preimplementation period, average monthly rate of PIM prescribing was 9.30% (95% CI, 8.82% to 9.78%). In the postimplementation period, PIM prescribing rate decreased to 8.62% (95% CI, 8.14% to 9.10%) (*P* < .01). During preimplementation, 1325 of 14 193 prescribed medications were PIMs, while only 1108 of 13 213 prescribed medications in postimplementation were PIMs.	Change of PIM rate by drug: antihistamines, 3.36% to 3.01% (difference, −0.35 pp [95% CI, −0.42 to −0.28 pp]); skeletal muscle relaxants, 3.08% to 2.36% (difference, −0.72 pp [95% CI, −0.82 to −0.62 pp]); benzodiazepines, 1.91% to 1.30% (difference, −0.61 pp; [95% CI, −0.70 to −0.62 pp]); antispasmodic, 0.32% to 0.25% (difference, −0.07 pp [95% CI, −0.10 to −0.04 pp]); nonsteroidal anti-inflammatory drug, 0.32% to 0.26% (difference, −0.06 pp [95% CI, −0.09 to −0.03 pp]).	
Moss et al,^48^ 2019 (North Carolina, United States)	February 2013 to December 2014	5662	Mean, ≥65 y	Not reported	Prospective cohort pre-post intervention	Academic detailing (AGS 2012 Beers Criteria)	Untrained resident cohort	Resident cohort who received educational intervention was less likely to prescribe a PIM compared with untrained resident cohort (RR, 0.73 [95% CI, 0.62 to 0.85]; *P* < .001).	PIM by residents: cyclobenzaprine: RR, 0.41 (95% CI, 0.30 to 0.57); *P* < .001; naproxen: RR, 0.23 ([95% CI, 0.14 to 0.38); *P* < .001; ibuprofen: RR, 0.23 (95% CI, 0.14 to 0.39); *P* < .001; hydroxyzine: RR, 0.75 (95% CI, 0.38 to 1.40); *P* = .38; diphenhydramine: RR, 0.56 (95% CI, 0.28 to 1.15); *P* = .11; methocarbamol: RR, 0.59 (95% CI, 0.29 to 1.2); *P* = .14.	
O’Connor et al,^50^ 2021 (United Kingdom)	September 2017 to October 2018	226	Mean, ≥65 y	43%	Prospective pre-post intervention	Plan-Do-Study-Act: spreading awareness via 3 presentations, 5 posters, and 1 email	Prescribing patterns before intervention	Reduction in the number of patients with ≥1 prescribing error (37% to 26%; *P* = .04). Proportion of patients with ≥1 STOPP error reduced from 17.5% to 9.0% (*P* = .03). Reduction in proportion of patients with ≥1 START error was not significant (24.6% to 20.7%; *P* = .24).	NA	
Stevens et al,^5^ 2017 (United States, multisite)	NA	Not reported	Mean, ≥65 y	Not reported	Prospective pre-post intervention comparison	EQUIPPED implementation: multicomponent QI initiative combining education, electronic clinical decision support, and individual clinician feedback	At least 6 mo prior to EQUIPPED implementation	Change in mean (SD) PIM pre-post intervention by site: site 1, 11.9 (1.8) vs 5.1 (1.4); *P* < .001; site 2, 8.2 (0.8) vs 4.5 (1.0); *P* < .001; site 3, 8.9 (1.9) vs 6.1 (1.7); *P* = .007; site 4, 7.4 (1.7) vs 5.7 (0.8); *P* = .04.	NA	
Vandenberg et al,^51^ 2024 (United States, multisite)	July 2018 to July 2021	Not reported	Mean, ≥65 y	Not reported	Prospective pre- and post-intervention comparison	EQUIPPED: initial group education on geriatric prescribing principles and training in how to access order sets, a 1:1 training session with a site champion, and monthly audit and feedback with peer benchmarking for 12 months	Pre-implementation	The proportion of PIMs at all 4 sites decreased significantly from pre- to post-EQUIPPED: at traditional site 1 from 8.9% (95% CI, 8.1% to 9.6%) to 3.6% (95% CI, 3.6% to 9.6%) (*P* < .001); at spread site 1 from 12.2% (95% CI, 11.2% to 13.2%) to 7.1% (95% CI, 6.1% to 8.1%) (*P* < .001); at spread site 2 from 11.3% (95% CI, 10.1% to 12.6%) to 7.9% (95% CI, 6.4% to 8.8%) (*P* = .045); and at spread site 3 from 16.2% (95% CI, 14.9% to 17.4%) to 11.7% (10.3% to 13.0%) (*P* < .001).	Time to implement was equivalent at all sites across both models. Interview data, reflecting a wide scope of responsibilities for the champion at the traditional site and a narrow scope at the spread sites, indicated disproportionate barriers to engagement at the spoke sites.	
Vaughan et al,^49^ 2021 (Georgia, United States)	NA	Not reported	Mean, ≥65 y	Not reported	Prospective pre-post intervention comparison	EQUIPPED implementation: multicomponent QI initiative combining education, electronic clinical decision support, and individual clinician feedback	6-month Period before EQUIPPED implementation	Pre-post monthly PIM proportion by site: site 1: 5.6% (95% CI, 5.0 to 6.3) vs 5.1% (95% CI, 4.7 to 5.5); *P* = .02; site 2: 5.8% (95% CI, 5.0 to 6.6) vs 5.4% (95% CI, 4.8 to 6.0), *P* = .62; site 3: 7.3% (95% CI, 6.4 to 9.2) vs 7.5% (95% CI, 6.6 to 8.4); *P* = .64.	In exploratory analyses, the proportion of benzodiazepine prescriptions decreased across all sites from approximately 17% of PIMs at baseline to 9.5% to 12% after implementation, although not all reached statistical significance.	
Vaughan et al,^52^ 2023 (United States, multisite)	October 2019 to September 2021	Not reported	Mean, ≥65 y	Not reported	Prospective parallel cluster RCT	Dashboard-based clinician feedback	Traditional personnel-intensive clinician feedback involving academic detailing delivered 1:1 by an EQUIPPED champion	During a 6-month baseline period, the academic detailing and dashboard sites had similar PIM prescribing rates of 8.01% for academic detailing vs 8.04% for dashboard (*P* = .90). Comparing 12 months of prescribing data after EQUIPPED implementation, the academic detailing group significantly improved PIM prescribing (7.07%) compared with the dashboard group (8.10%) (OR, 1.14 [95% CI, 1.08 to 1.22]; *P* < .001).	Within the groups, 2 of 4 academic detailing sites demonstrated statistically significant reductions in PIM prescribing; 1 of 4 dashboard sites achieved nearly 50% relative reduction in PIM prescribing.	
**Computerized CDSS studies**										
Griffey et al,^53^ 2012 (Massachusetts, United States)	July 2006 to January 2007	1407	Mean, 75 y	39%	Prospective controlled trial	Computerized CDSS: ED order entry system	On vs off period	Majority of recommendations for alternate medications were declined (49/53 [92.5%]). More orders were consistent with dosing recommendations during on periods (403/1283 [31%]) than off periods (256/1115 [23%]) (*P* < .001). Overall agreement with recommendations was low for on vs off periods: 403/1283 (31% [95% CI, 29% to 34%]) vs 256/1115 (23% [95% CI, 21% to 26%]).	The rate of ADEs was lower during on (8/237 [3.4%]) compared with off (31/436 [7.1%]) periods (*P* = .02). Remaining secondary outcomes showed no difference. Acceptance rate of computer recommended appropriate dose for individual medications, on vs off: benzodiazepines, 29% vs 24%; *P* = .29; nonsteroidal anti-inflammatory drugs, 19% vs 11%; *P* = .04; opiates, 36% vs 26%; *P* < .001; sedatives-hypnotics, 15% vs 12%; *P* = .64; and adverse events, 8 (3% [95% CI, 1% to 6%]) vs 31 (7% [95% CI, 5% to 9%]); *P* = .02.	
Kim et al,^54^ 2017 (Washington, United States)	May 2015 to April 2016	1946	Mean, 73 y	25%	Prospective before-and-after intervention study	CPOE adjustment based on established pharmacy guidelines and expert consensus: default geriatric dosing	Before vs after CPOE template modification	Significant improvement in the rate of recommended dose administration of all medications of interest before vs after CPOE template modification (27.3% vs 32.5%; *P* < .001).	Medications of interest: opioids, 29.0% vs 35.2%; *P* < .001; benzodiazepines, 20.1% vs 25.5%; *P* = .08; and nonsteroidal anti-inflammatory drugs, 18.4% vs 17.0%; *P* = .76.	
Liu et al,^55^ 2019 (Taiwan)	December 2017 to October 2018	911	78 y	49.8%	Prospective, observational pre-post	Automatic screening by the computer-based medication reconciliation and integration system	Preintervention group (historical control)	Number of medications was reduced from a mean (SD) of 12.5 (2.7) to 6.9 (3.0) in postintervention period in patients with major polypharmacy (*P* < .001).	Proportions of major polypharmacy and PIM were lower in the postintervention than in the preintervention period (−79.4% vs −65.3%; *P* < .001, and −67.5% vs −49.1%; *P* < .001, respectively).	
Terrell et al,^56^ 2009 (Indiana, United States)	January 2005 to July 2007	5162 Visits	Mean, 74 y	35%	Prospective RCT	Computer-assisted decision support that advised against use of 9 PIMs and recommended safer substitute therapies	Control: did not receive the decision support, but the computer system tracked their prescribing	Decision support provided 114 times to intervention physicians, who accepted 49 of the recommendations (43%). Intervention physicians prescribed ≥1 inappropriate medications during 2.6% of ED visits, compared with 3.9% visits managed by control physicians (OR, 0.55 [95% CI, 0.34 to 0.89]; *P* = .02; aRR, 1.3% [95% CI, 0.4% to 2.3%]).	Proportion of all prescribed medications that were potentially inappropriate decreased from 103 (5.4%) to 69 (3.4%) (OR, 0.59 [95% CI, 0.41 to 0.85]; *P* = .006; ARR, 2.0% [95% CI, 0.7% to 3.3%]).	
**FRID ** **review studies**										
Boyé et al,^57^ 2017 (the Netherlands)	NA	612	Mean, 76 y	38%	Prospective multicenter RCT	Withdrawal of FRIDs by research physician via fall-related assessment, including medical history and medication use (IMPROVeFALL)	Usual care	Intervention did not have a significant association time to first fall HR, 1.17 (95% CI, 0.89 to 1.54).	Time to second fall: HR, 1.19, (95% CI, 0.78 to 1.82); time to first fall-related GP consultation: HR, 0.66 (95% CI, 0.42 to 1.06); time to first fall-related ED visit: HR, 0.85 (95% CI, 0.43 to 1.68).	
Polinder et al,^58^ 2016 (the Netherlands)	October 2008 to October 2011	612	Mean, 76 y	38%	Prospective multicenter RCT	Systematic FRIDs assessment by research assistant combined with FRIDs withdrawal or modification in consultation with geriatrician or prescribing physician (IMPROVeFALL)	Care as usual for fall injuries and structured medication assessment	Total fall-related health care costs did not differ significantly between the intervention group and the control group (€2204 vs €2285). The withdrawal of FRIDs reduced medication costs a mean of €38 per participant. The control group had a greater decline in EuroQol-5D utility score during the 12-mo follow-up than the intervention group (*P* = .02).	NA	
Tan et al,^59^ 2018 (Malaysia)	2012 to February 2016	268	Mean, 75 y	33%	Pragmatic RCT	Individually tailored multifactorial interventions, including modified Otago exercise program, visual intervention, HOMEFAST home environmental modification, cardiovascular intervention, medication review, and falls education	Control group received conventional treatment	Fall recurrence did not differ between intervention and control groups at 12 months (70.5% vs 70.1%; RR, 1.04 [95% CI, 0.61 to 1.75]; *P* = .89).	Rate of fall: RR, 1.16 (95% CI, 0.85 to 1.58); time to first fall: HR, 0.95 (95% CI, 0.78 to 1.52); mortality rate: RR, 0.90 (95% CI, 0.34 to 2.40).	

Abbreviations: ADE, adverse drug event; AGS, American Geriatrics Society; aIRR, adjusted incidence rate ratio; aOR, adjusted odds ratio; ARR, adjusted rate ratio; BPMH, best-possible medication history; CDSS, clinical decision support system; CPOE, computerized physician order entry; CPS, clinical pharmacy specialist; ED, emergency department; EHR, electronic health record; EM, emergency medicine; EQUIPPED, Enhancing Quality of Prescribing Practices for Older Adults Discharged From the Emergency Department; FRID, fall risk–increasing drug; GPs, general practitioners; HR, hazard ratio; LOS, length of stay; NA, not applicable; OAEM, older adult emergency medicine; OR, odds ratio; PCP, primary care physician; PIM, potentially inappropriate medication; pp, percentage point; PPMC, partnered pharmacist medication charting; QI, quality improvement; RCT, randomized clinical trial; RR, rate ratio; STOPP/START, Screening Tool of Older Person’s Prescriptions/Screening Tool to Alert Doctors to Right Treatment.

### Clinical Pharmacist Reviews

#### Overview

Nine studies,^36,37,38,39,40,41,42,43,44^ enrolling 28 360 participants, evaluated a pharmacist-led medication review within the ED to identify PIMs and provide recommendations. The mean or median age in the studies was 70 years and older, and the sample sizes ranged from 110 to 10 807 participants.

#### Medication Recommendations

Briggs et al^36^ reported that clinicians adopted 49% of pharmacists’ recommendations. Marks et al^37^ examined patient adoption of pharmacists’ recommendations after a motivational interviewing–based intervention. They found patients were able to partially or fully uptake 81% (95% CI, 67%-91%) of advisory recommendations, such as “discuss further with prescriber,” and 64% (95% CI, 53%-74%) of recommendations with specific actions, such as “decrease dose.”^37^

#### Hospital Admission and LOS

Rates of hospital admission and hospital LOS were calculated in 2 studies. Briggs et al^36^ found odds of admission were lower among patients receiving clinical pharmacist review (OR, 0.68 [95% CI, 0.53-0.87]; *P* = .002). However, Hohl et al^38^ found in-hospital pharmacist-led medication review was not associated with admissions.^38^ Neither found a significant association between the program and hospital LOS.^36,38^

#### Subsequent Health Care Utilization

In 5 studies,^36,38,39,40,41^ pharmacist-based medication review was not associated with reduced ED revisits, readmissions, or primary care visits. In Clementz et al,^42^ pharmacist-led medication review was associated with significantly lower rates of unplanned rehospitalizations (within 72 hours: OR, 0.24 [95% CI, 0.06-0.94]; within 30 days: OR, 0.45 [95% CI, 0.26-0.95]; within 90 days: OR, 0.45 [95% CI, 0.26-0.79]). However, this study was judged to have serious ROB. Marks et al^37^ similarly found pharmacist-led motivational interviewing was associated with reduced repeat all-cause ED visits (adjusted incidence rate ratio [aIRR], 0.47 [95% CI, 0.29-0.74]).^37^ Conversely, Shaw et al^43^ found patients receiving pharmacist medication reconciliation and review were more likely to have a 30-day return visit (adjusted OR, 1.34 [95% CI, 1.02-1.76]).^43^

#### PIM Deprescribing

Two studies^41,44^ found pharmacist-performed medication review and reconciliation were associated with significantly reduced PIM use at ED discharge. Atey et al^44^ found use of at least 1 PIM on ED departure was significantly lower for the intervention group than comparison groups (*P* = .04). Similarly, Jovevski et al^41^ found that case rate of PIM deprescribing in a preintervention group was 11.1% vs 57.1% in a postintervention (pharmacist-led medication reconciliation) group (*P* < .001).

### Geriatrician Teleconsultation

Matz et al^45^ evaluated geriatrician teleconsultation and pharmacological recommendations among 50 ED patients aged 70 years and older with an Identification of Seniors at Risk Score of 2 or greater.^45^ There was a higher frequency of recommendations, including drug discontinuation (*P* < .001), drug initiation (*P* = .004), or dose adjustment (*P* = .001), via geriatric telemedicine compared with standard ED treatment. Total medications per patient and number of PIMs were lower with geriatrician teleconsultation (*P* < .001). ED physicians more frequently made recommendations for immediate drug therapy than geriatricians (*P* = .04).

### Clinician Educational Interventions

#### Geriatric Training

Eight studies evaluated clinician educational interventions and prescribing PIMs to older adults in the ED.^5,46,47,48,49,50,51,52^ Two^46,48^ evaluated the association of geriatric training and academic detailing with resident physician prescribing patterns. Biese et al^46^ found a geriatric curriculum was not associated with a change the number of older ED patients receiving sedation, while Moss et al^48^ found resident physicians were less likely to prescribe a PIM to older ED patients after academic detailing (rate ratio, 0.73 [95% CI, 0.62-0.85]). Both were deemed to have serious ROB, limiting interpretability.

#### PIM Prescribing

Five studies^5,47,49,51,52^ across 22 US sites evaluated the Enhancing Quality of Prescribing Practices for Older Veterans Discharged From the ED (EQUIPPED) program, including ED clinician education through academic detailing, computerized CDSS (medication order sets), and feedback on prescribing practices. Compared with the preimplementation period, there were significantly lower rates of PIM prescribing among older adults discharged from the ED in the majority of sites (15 of 22 [68%]), ranging from a 0.5% to 6.8% reduction overall, calculated in Stevens et al^5^ as an absolute reduction of more than 50 PIMs monthly.^5^ Goldberg et al^47^ found lower PIM rates for benzodiazepines (change, −0.61% [95% CI, −0.70% to −0.62%]), skeletal muscle relaxants (change, −0.72%, [95% CI, −0.82% to −0.62%]), and antihistamines (change, −0.35% [95% CI, −0.42% to −0.28%]).^47^

#### Prescribing Errors

A Plan-Do-Study-Act cycle study examined whether presentations, posters, and emails were associated with reduced prescribing errors based on STOPP/START.^50^ O’Connor et al^50^ found a reduction in the number of patients with at least 1 prescribing error (37% to 26%; *P* = .04) and proportion of patients with at least 1 STOPP error (17.5% to 9.0%; *P* = .03).^50^

### Computerized CDSS

#### Overview

Four studies evaluated a computerized CDSS for medication and dosing appropriateness.^53,54,55,56^ These included a total of 9426 patients (mean ages, 73-78 years), with sample sizes ranging from 911 to 5162.

#### PIM Deprescribing

Liu et al^55^ utilized a computer-based, pharmacist-assisted medication reconciliation and integration system that was associated with a reduction in major polypharmacy (≥10 medications; −79.4% vs −65.3%; *P* < .001). It was also associated with a reduction in PIMs (−67.5% vs −49.1%; *P* < .001).^55^

#### PIM Ordering and Prescribing

In 3 of 4 studies that evaluated PIM ordering and prescribing as an outcome, computerized CDSS was significantly associated with a reduced proportion of PIM usage.^53,54,56^ Terrell et al^56^ found computerized CDSS for computerized physician order entry (CPOE) was associated with a lower proportion of PIM prescriptions, changing from 5.4% to 3.4% (OR, 0.59 [95% CI, 0.41-0.85]; *P* = .006).^56^ Griffey et al^53^ and Kim et al^54^ found computerized CDSS was associated with improved CPOE adherence to recommended geriatric dose administration rate for opioids (Griffey et al^53^: 36% vs 26% [control], *P* < .001; Kim et al^54^: 29.0% [control] vs 35.2%, *P* < .001) but differed in outcomes for appropriate dosing of benzodiazepines and nonsteroidal anti-inflammatory medications.^53,54^

#### ADEs

Griffey et al^53^ reported the rate of ADEs was lower with use of computerized CDSS compared with usual care. For computerized CDSS, the rate was 3.4%, while the usual care rate was 7.1% (*P* = .02).

### FRID Reviews

#### Overview

Three RCTs^57,58,59^ focused on reducing FRIDs with medication review as part of multifactorial fall prevention programs among older patients presenting to the ED after falls. These studies included a total of 880 patients (mean ages, 75-76 years), with sample sizes from 268 to 612.

#### Fall Rates

Boye et al^57^ and Tan et al^59^ reported no difference in time to first fall or rates of falls and fall recurrence at 12 months between the intervention and control group. However, Marks et al^37^ found a pharmacist-led motivational interviewing–based intervention was associated with reduced repeat fall-related ED visits (aIRR, 0.34 [95% CI, 0.15-0.76]). Furthermore, Polinder et al^58^ evaluated health-related quality of life measured with the EuroQol Quality of Life scale and found the control group had a greater decline after 12 months (*P* = .02) compared with intervention.^58^

### Meta-Analysis

After reviewing the consistency of intervention, outcome, and effect, we conducted 7 meta-analyses. Given fewer than 3 included studies, we were unable to assess publication bias.^61^

#### Clinical Pharmacist Review and Hospital LOS

Average difference in hospital LOS for the 2 included studies^36,38^ ranged from −0.05 to 0.00 days. Random-effects meta-analysis showed the combined average difference in LOS across studies was −0.03 days (95% CI, −4.19 to 4.12 days; *P* = .99) (eFigure 2A in Supplement 1). The funnel plot showed very minimal heterogeneity (Cochran *Q* = 0.0001; *df* = 1; *P* = .99; *I*^2^ = 0.00%) (eFigure 1A in Supplement 1).

#### Clinical Pharmacist Review and Hospital Admission

The ORs for hospital admission rates for the 2 included studies^36,38^ ranged from 0.68 to 1.05. Random-effects meta-analysis showed the combined OR was 0.86 (95% CI, 0.56-1.31; *P* = .48) (eFigure 2B in Supplement 1). The funnel plot showed substantial heterogeneity (Cochran *Q* = 10.603; *df* = 1; *P* = .001; *I*^2^ = 90.57%) (eFigure 1B in Supplement 1).

#### Clinical Pharmacist Review and PIM Deprescribing

**Figure 4.**
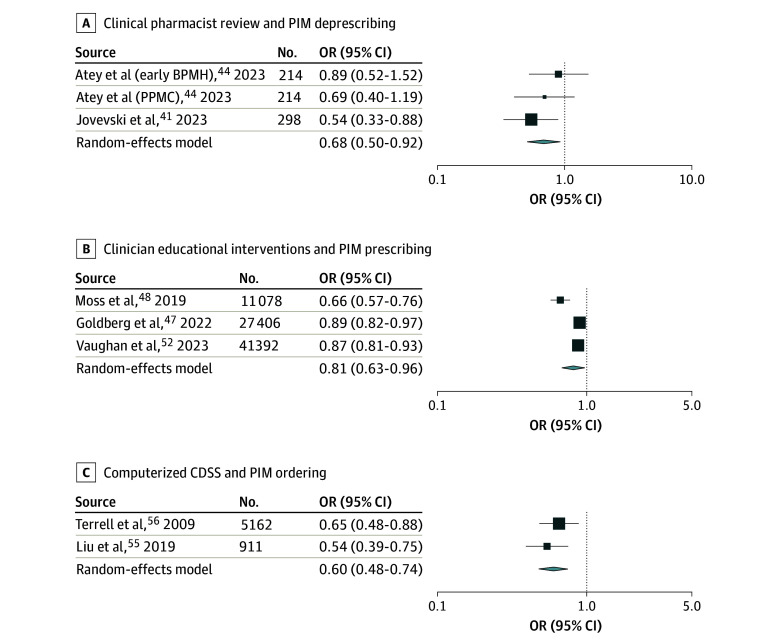
Results of Random-Effects Meta-Analysis Models The size of the boxes (symbols) is proportional to the sample size. BPMH indicates best-possible medication history; CDSS, clinical decision support systems; OR, odds ratio; PIM, potentially inappropriate medications; PPMC, partnered pharmacist medication charting.

The OR for PIM deprescribing for 2 studies^41,44^ ranged from 0.33 to 0.52. Random-effects meta-analysis showed the combined OR was 0.68 (95% CI, 0.50-0.92; *P* = .01), implying a 32% reduction of PIMs with clinical pharmacist review (Figure 4A). The funnel plot showed very minimal heterogeneity (Cochran *Q* = 1.808; *df* = 2; *P* = .41; *I*^2^ = 0.00%) (eFigure 1C in Supplement 1).

#### Clinician Educational Interventions and PIM Prescribing

The ORs for PIM prescribing for the 3 included studies^47,48,52^ ranged from 0.66 to 0.89. Random-effects meta-analysis showed the combined OR was 0.81 (95% CI, 0.68-0.96; *P* = .02), implying a 19% reduction of PIMs with educational interventions (Figure 4B). The funnel plot showed substantial heterogeneity (Cochran *Q* = 13.057; *df* = 2; *P* = .002; *I*^2^ = 90.69%) (eFigure 1D in Supplement 1).

#### Computerized CDSS and PIM Ordering

The OR for PIM ordering for the 2 included studies^55,56^ ranged from 0.54 to 0.65. Random-effects meta-analysis showed the combined OR was 0.60 (95% CI, 0.48-0.74; *P* < .001), implying a 40% reduction of PIM ordering with computerized CDSS (Figure 4C). The funnel plot showed minimal heterogeneity (Cochran *Q* = 0.667; *df* = 1; *P* = .41; *I*^2^ = 0.00%) (eFigure 1E in Supplement 1).

#### FRID Review and Fall Recurrence at 12 Months

The ORs for fall recurrence at 12 months for the 2 included studies^57,59^ ranged from 1.04 to 1.14. Random-effects meta-analysis showed the combined OR was 1.11 (95% CI, 0.83-1.48; *P* = .48) (eFigure 2C in Supplement 1). The funnel plot showed minimal heterogeneity (Cochran *Q* = 0.081; *df* = 1; *P* = .78; *I*^2^ = 0.00%) (eFigure 1F in Supplement 1).

#### FRID Review and Time to First Fall

The HRs for time to first fall for the 2 included studies^57,59^ ranged 0.95 to 1.17. Random-effects meta-analysis showed the combined HR was 1.03 (95% CI, 0.84-1.26; *P* = .78) (eFigure 2D in Supplement 1). The funnel plot showed minimal heterogeneity (Cochran *Q* = 1.520; *df* = 1; *P* = .22; *I*^2^ = 34.22%) (eFigure 1G in Supplement 1).

## Discussion

Overall, our systematic review and meta-analyses found certain ED-based geriatric medication programs were associated with improved PIM deprescribing and reduced PIM ordering and prescribing among older adults. Specifically, clinical pharmacist review or geriatric teleconsultation was associated with improved PIM deprescribing but was not associated with improved hospital admission rates or LOS. Furthermore, clinician educational interventions and computerized CDSS were associated with reduced PIM ordering and prescribing. Finally, FRID review as part of ED-based fall prevention programs were not associated with reduced time to first fall or fall recurrence at 12 months.

Our systematic review identified a positive impact of clinical pharmacists and geriatrician teleconsultation in ED-based geriatric medication safety programs.^36,37,38,39,40,42,43^ There is broad support for ED clinical pharmacist services from emergency medicine, toxicology, and pharmacy organizations in the US.^62,63,64^ Our meta-analysis demonstrated that clinical pharmacist review was not associated with decreased hospital admission or LOS, but 2 studies^41,44^ showed a 32% reduction in PIMs from deprescribing. Furthermore, pharmacists were associated with improved outcomes, such as medication safety recommendations and unplanned rehospitalizations, in our included studies, but likely did not affect subsequent health care utilization.^36,37,38,39,40,41,42^ Additionally, 1 study^45^ demonstrated geriatrician teleconsultations in the ED were associated with enhanced deprescribing of PIMs compared with changes made by ED physicians. However, barriers to medication deprescribing include patients’ and physicians’ unwillingness, fear of negative consequences, lack of time, and poor communication between multiple prescribers.^65,66^

Three EQUIPPED studies showed a 19% reduction of PIM prescribing through ED clinician education. Our meta-analysis supports academic detailing to enhance quality of prescribing practices for older adults in the ED.^67,68^ However, academic detailing requires effort to provide individualized clinician feedback and audit prescribing rates. Furthermore, academic detailing requires buy-in from prescribers and could cause resentment around prescription patterns being tracked.^69,70^ Sustainability of these interventions and long-term effects have yet to be determined.

Two computerized CDSS studies showed 40% reduction of PIM ordering, similar to other studies reporting positive impact of computerized CDSS to decrease clinician cognitive load.^71^ Use of computerized CDSS in routine ED practice may be a practical option for reducing adverse events related to high-risk medications for older adults, especially given widespread use of electronic health records (EHR). While there may be up-front costs to implementing EHR-based medication safety programs, such programs could be shared across EDs and do not require additional staffing resources, such as pharmacist and geriatrician consultations or academic detailing.^5,47,49,67^ Conversely, drawbacks to EHR interventions include clinician fatigue with best practice alerts.^72,73^

Finally, multifactorial fall prevention programs that included medication review for FRIDs were not associated with reduced time to first fall or fall recurrence at 12 months; however, pharmacist-led motivational interviewing–based interventions were associated with reduced repeat fall-related ED visits, and FRID review was associated with reduced functional decline at 12 months.^37,57,58,59^ Future studies are needed to determine which interventions are effective at reducing future falls.

Our findings from this systematic review and meta-analysis will be combined with findings from another systematic review on comparative safety of sedating medications for agitation among older adults in a subsequent article. These will inform our recommendations on implementing ED-based geriatric medication safety programs in the upcoming Geriatric ED Guidelines version 2.0 following the Grading of Recommendations, Assessment, Development, and Evaluation methods.^74,75,76^

### Limitations

There are several limitations to this systematic review and meta-analysis. We focused on ED-based interventions for older adults. There may be other interventions for geriatric patients that were excluded because they did not focus on older adults or were not based in the ED, such as a comprehensive intervention bundle using the Drug Burden Index to facilitate deprescribing.^77^ As we excluded studies that were not RCTs or observational cohort studies, we may have missed other potentially effective interventions. The limited number of studies with data available for meta-analysis prevented assessment of publication bias. As only 1 study had low ROB, generalizability of study findings is limited, and results of our meta-analysis should be interpreted with caution. Although most studies assessing outcome measures did not find a clear benefit to patients, many showed significant improvement in process measures, such as PIM deprescribing, prescribing, and ordering. Outcome measures often require a longer timeframe and are influenced by multiple factors beyond the specific process being measured, making it challenging to definitively attribute changes in the outcome to the process alone. Future studies on ED-based geriatric medication safety programs should evaluate appropriate patient-centered outcomes, which will be critical for implementation.

## Conclusions

In this systematic review and meta-analysis of ED-based geriatric medication safety programs, involvement of a multidisciplinary team, including clinical pharmacists and/or geriatricians, was associated with improved PIM deprescribing. Furthermore, computerized CDSS, alone or in combination with ED clinician education, was associated with enhanced geriatric ordering and prescribing practices. Although these studies demonstrated that the interventions were associated with improved process measures, future studies will be needed to determine whether they impact patient-centered outcomes, such adverse events and health care utilization. These findings will inform the Geriatric ED Guidelines version 2.0 update.

## Notes

Supplement 1. **eMethods.** Search Criteria and Strategies **eFigure 1.** Funnel Plots and Fail-Safe N Calculation Using Rosenberg Approach of Meta-Analyses **eFigure 2.** Results of Random-Effects Meta-Analysis Models **eFigure 3.** Extracted Data From Source **eTable.** Included Studies Inclusion and Exclusion Criteria

Supplement 2.
Nonauthor Collaborators

Supplement 3.
Data Sharing Statement

## References

[1] Cairns C, Kang K. National Hospital Ambulatory Medical Care Survey: 2020 emergency department summary tables. National Center for Health Statistics. Accessed February 5, 2025. https://www.cdc.gov/nchs/data/nhamcs/web_tables/2020-nhamcs-ed-web-tables-508.pdf

[2] Kim M, Mitchell SH, Gatewood M, . Older adults and high-risk medication administration in the emergency department. Drug Healthc Patient Saf. 2017;9:105-112. doi:10.2147/DHPS.S143341 29184448 PMC5685141

[3] Theou O, Campbell S, Malone ML, Rockwood K. Older adults in the emergency department with frailty. Clin Geriatr Med. 2018;34(3):369-386. doi:10.1016/j.cger.2018.04.003 30031422

[4] Budnitz DS, Pollock DA, Weidenbach KN, Mendelsohn AB, Schroeder TJ, Annest JL. National surveillance of emergency department visits for outpatient adverse drug events. JAMA. 2006;296(15):1858-1866. doi:10.1001/jama.296.15.1858 17047216

[5] Stevens M, Hastings SN, Markland AD, . Enhancing Quality of Provider Practices for Older Adults in the Emergency Department (EQUiPPED). J Am Geriatr Soc. 2017;65(7):1609-1614. doi:10.1111/jgs.14890 28388818

[6] Xing XX, Zhu C, Liang HY, . Associations between potentially inappropriate medications and adverse health outcomes in the elderly: a systematic review and meta-analysis. Ann Pharmacother. 2019;53(10):1005-1019. doi:10.1177/1060028019853069 31129978

[7] Mekonnen AB, Redley B, de Courten B, Manias E. Potentially inappropriate prescribing and its associations with health-related and system-related outcomes in hospitalised older adults: a systematic review and meta-analysis. Br J Clin Pharmacol. 2021;87(11):4150-4172. doi:10.1111/bcp.14870 34008195 PMC8597090

[8] Passarelli MC, Jacob-Filho W, Figueras A. Adverse drug reactions in an elderly hospitalised population: inappropriate prescription is a leading cause. Drugs Aging. 2005;22(9):767-777. doi:10.2165/00002512-200522090-00005 16156680

[9] Lau DT, Kasper JD, Potter DE, Lyles A, Bennett RG. Hospitalization and death associated with potentially inappropriate medication prescriptions among elderly nursing home residents. Arch Intern Med. 2005;165(1):68-74. doi:10.1001/archinte.165.1.68 15642877

[10] Shehab N, Lovegrove MC, Geller AI, Rose KO, Weidle NJ, Budnitz DS. US emergency department visits for outpatient adverse drug events, 2013-2014. JAMA. 2016;316(20):2115-2125. doi:10.1001/jama.2016.16201 27893129 PMC6490178

[11] Kantor ED, Rehm CD, Haas JS, Chan AT, Giovannucci EL. Trends in prescription drug use among adults in the United States from 1999-2012. JAMA. 2015;314(17):1818-1831. doi:10.1001/jama.2015.13766 26529160 PMC4752169

[12] Hastings SN, Schmader KE, Sloane RJ, . Quality of pharmacotherapy and outcomes for older veterans discharged from the emergency department. J Am Geriatr Soc. 2008;56(5):875-880. doi:10.1111/j.1532-5415.2008.01648.x 18341519

[13] Delara M, Murray L, Jafari B, . Prevalence and factors associated with polypharmacy: a systematic review and Meta-analysis. BMC Geriatr. 2022;22(1):601. doi:10.1186/s12877-022-03279-x 35854209 PMC9297624

[14] Martin CB, Hales CM, Gu Q, Ogden CL. Prescription drug use in the United States, 2015-2016. NCHS Data Brief. 2019;(334):1-8. 31112126

[15] Beers MH, Ouslander JG, Rollingher I, Reuben DB, Brooks J, Beck JC; UCLA Division of Geriatric Medicine. Explicit criteria for determining inappropriate medication use in nursing home residents. Arch Intern Med. 1991;151(9):1825-1832. doi:10.1001/archinte.1991.00400090107019 1888249

[16] By the 2023 American Geriatrics Society Beers Criteria Update Expert Panel. American Geriatrics Society 2023 updated AGS Beers Criteria for potentially inappropriate medication use in older adults. J Am Geriatr Soc. 2023;71(7):2052-2081. doi:10.1111/jgs.18372 37139824 PMC12478568

[17] O'Mahony D, Cherubini A, Guiteras AR, et al. STOPP/START criteria for potentially inappropriate prescribing in older people: version 3. Eur Geriatr Med. 2023;14(4):625-632. doi:10.1007/s41999-023-00777-y PMC10447584 37256475

[18] Gallagher P, Ryan C, Byrne S, et al. STOPP (Screening Tool of Older Person's Prescriptions) and START (Screening Tool to Alert doctors to Right Treatment). Consensus validation. Int J Clin Pharmacol Ther. 2008;46(2):72-83. doi:10.5414/cpp46072 18218287

[19] LoCicero J. A Supplement to New Frontiers in Geriatrics Research: An Agenda for Surgical and Related Medical Specialties. American Geriatrics Society; 2007.

[20] Carpenter CR, Heard K, Wilber S, ; Society for Academic Emergency Medicine (SAEM) Geriatric Task Force. Research priorities for high-quality geriatric emergency care: medication management, screening, and prevention and functional assessment. Acad Emerg Med. 2011;18(6):644-654. doi:10.1111/j.1553-2712.2011.01092.x 21676064 PMC3117251

[21] Carpenter CR, Shah MN, Hustey FM, Heard K, Gerson LW, Miller DK. High yield research opportunities in geriatric emergency medicine: prehospital care, delirium, adverse drug events, and falls. J Gerontol A Biol Sci Med Sci. 2011;66(7):775-783. doi:10.1093/gerona/glr040 21498881 PMC3143344

[22] Centers for Medicare & Medicaid Services. Medicare and Medicaid programs and the Children’s Health Insurance Program; hospital inpatient prospective payment systems for acute care hospitals and the long-term care hospital prospective payment system and policy changes and fiscal year 2025 rates; quality programs requirements; and other policy changes. May 2, 2024. Accessed February 3, 2025. https://www.federalregister.gov/documents/2024/05/02/2024-07567/medicare-and-medicaid-programs-and-the-childrens-health-insurance-program-hospital-inpatient#open-comment2024.

[23] American College of Emergency Physicians; American Geriatrics Society; Emergency Nurses Association; Society for Academic Emergency Medicine; Geriatric Emergency Department Guidelines Task Force. Geriatric emergency department guidelines. Ann Emerg Med. 2014;63(5):e7-e25. doi:10.1016/j.annemergmed.2014.02.008 24746436

[24] Santangelo I, Ahmad S, Liu S, . Examination of geriatric care processes implemented in level 1 and level 2 geriatric emergency departments. J Geriatr Emerg Med. 2022;3(4). 36970655 10.17294/2694-4715.1041 PMC10035774

[25] Gardner K, Schwarz K, Pearson S, Jacknin G. Potentially inappropriate medication usage in older adults in a tertiary academic medical center emergency department. J Pharm Pract. 2022;35(6):892-897. doi:10.1177/08971900211016813 34000882

[26] Goldberg EM, Dresden SM, Carpenter CR. Clin-STAR corner: practice changing advances in prescribing for geriatric emergency department patients. J Am Geriatr Soc. 2023;71(12):3686-3691. doi:10.1111/jgs.18619 37801018 PMC12994110

[27] Liberati A, Altman DG, Tetzlaff J, . The PRISMA statement for reporting systematic reviews and meta-analyses of studies that evaluate health care interventions: explanation and elaboration. Ann Intern Med. 2009;151(4):W65-94. doi:10.7326/0003-4819-151-4-200908180-00136 19622512

[28] Sterne JA, Hernán MA, Reeves BC, . ROBINS-I: a tool for assessing risk of bias in non-randomised studies of interventions. BMJ. 2016;355:i4919. doi:10.1136/bmj.i4919 27733354 PMC5062054

[29] Sterne JAC, Savović J, Page MJ, . RoB 2: a revised tool for assessing risk of bias in randomised trials. BMJ. 2019;366:l4898. doi:10.1136/bmj.l4898 31462531

[30] Wan X, Wang W, Liu J, Tong T. Estimating the sample mean and standard deviation from the sample size, median, range and/or interquartile range. BMC Med Res Methodol. 2014;14:135. doi:10.1186/1471-2288-14-135 25524443 PMC4383202

[31] Hedges LV, Vevea JL. Fixed- and random-effects models in meta-analysis. Psychol Methods. 1998;3:486-504. doi:10.1037/1082-989X.3.4.486

[32] Hedges LV, Olkin I. Statistical Methods for Meta-Analysis. Academic Press; 1985.

[33] Rosenberg MS. The file-drawer problem revisited: a general weighted method for calculating fail-safe numbers in meta-analysis. Evolution. 2005;59(2):464-468. 15807430

[34] Sterne JA, Sutton AJ, Ioannidis JP, . Recommendations for examining and interpreting funnel plot asymmetry in meta-analyses of randomised controlled trials. BMJ. 2011;343:d4002. doi:10.1136/bmj.d4002 21784880

[35] Viechtbauer W. Conducting meta-analyses in R with the metafor package. J Stat Softw. 2010;36(3):1-48. doi:10.18637/jss.v036.i03

[36] Briggs S, Pearce R, Dilworth S, Higgins I, Hullick C, Attia J. Clinical pharmacist review: a randomised controlled trial. Emerg Med Australas. 2015;27(5):419-426. doi:10.1111/1742-6723.12451 26190132

[37] Marks SJ, Long S, Deirmenjian A, Goldberg EM. Patient adoption of pharmacist recommendations to older adults presenting to emergency department with falls: a secondary analysis of GAPcare. Acad Emerg Med. 2021;28(11):1321-1324. doi:10.1111/acem.14302 34033186 PMC8613298

[38] Hohl CM, Partovi N, Ghement I, . Impact of early in-hospital medication review by clinical pharmacists on health services utilization. PLoS One. 2017;12(2):e0170495. doi:10.1371/journal.pone.0170495 28192477 PMC5305222

[39] Kitchen SA, McGrail K, Wickham ME, Law MR, Hohl CM. Emergency department-based medication review on outpatient health services utilization: interrupted time series. BMC Health Serv Res. 2020;20(1):254. doi:10.1186/s12913-020-05108-6 32216791 PMC7098150

[40] Santolaya-Perrín R, Calderón-Hernanz B, Jiménez-Díaz G, . The efficacy of a medication review programme conducted in an emergency department. Int J Clin Pharm. 2019;41(3):757-766. doi:10.1007/s11096-019-00836-0 31028596

[41] Jovevski JJ, Smith CR, Roberts JL, . Implementation of a compulsory clinical pharmacist-led medication deprescribing intervention in high-risk seniors in the emergency department. Acad Emerg Med. 2023;30(4):410-419. doi:10.1111/acem.14699 36794336

[42] Clementz A, Jost J, Lacour A, . Effect of clinical pharmacy services in an older adult emergency medicine unit on unplanned rehospitalization of older adults admitted for falls: MUPA-PHARM study. J Am Med Dir Assoc. 2019;20(8):947-948. doi:10.1016/j.jamda.2019.02.029 31353043

[43] Shaw PB, Delate T, Lyman A Jr, . Impact of a clinical pharmacy specialist in an emergency department for seniors. Ann Emerg Med. 2016;67(2):177-188. doi:10.1016/j.annemergmed.2015.06.022 26211427

[44] Atey TM, Peterson GM, Salahudeen MS, Wimmer BC. The impact of partnered pharmacist medication charting in the emergency department on the use of potentially inappropriate medications in older people. Front Pharmacol. 2023;14:1273655. doi:10.3389/fphar.2023.1273655 38026998 PMC10664652

[45] Matz O, Villa L, Lecce C, . Implementation of a telemedicine geriatric co-evaluation in the emergency department: a prospective pilot study. Swiss Med Wkly. 2021;151:w20500. doi:10.4414/smw.2021.20500 34000061

[46] Biese KJ, Roberts E, LaMantia M, . Effect of a geriatric curriculum on emergency medicine resident attitudes, knowledge, and decision-making. Acad Emerg Med. 2011;18(suppl 2):S92-S96. doi:10.1111/j.1553-2712.2011.01170.x 21999564

[47] Goldberg EM, Lin TR, Cunha CB, Mujahid N, Davoodi NM, Vaughan CP. Enhancing the quality of prescribing practices for older adults discharged from the emergency department in Rhode Island. J Am Geriatr Soc. 2022;70(10):2905-2914. doi:10.1111/jgs.17955 35809226 PMC9588533

[48] Moss JM, Bryan WE III, Wilkerson LM, . An interdisciplinary academic detailing approach to decrease inappropriate medication prescribing by physician residents for older veterans treated in the emergency department. J Pharm Pract. 2019;32(2):167-174. doi:10.1177/0897190017747424 29277130 PMC6533068

[49] Vaughan CP, Hwang U, Vandenberg AE, . Early prescribing outcomes after exporting the EQUIPPED medication safety improvement programme. BMJ Open Qual. 2021;10(4):e001369. doi:10.1136/bmjoq-2021-001369 34750188 PMC8576471

[50] O’Connor J, Adabavazeh B, Choi H, . Use of the STOPP and START criteria to address polypharmacy for elderly patients in University Hospital Lewisham clinical decisions unit. Hong Kong J Emerg Med. 2021;28(2):79-84. doi:10.1177/1024907919849358

[51] Vandenberg AE, Hwang U, Das S, . Scaling the EQUIPPED medication safety program: traditional and hub-and-spoke implementation models. J Am Geriatr Soc. 2024;72(7):2184-2194. doi:10.1111/jgs.18746 38259070

[52] Vaughan CP, Burningham Z, Kelleher JL, ; The EQUIPPED VA Implementation QI Group. A cluster-randomized trial of two implementation strategies to deliver audit and feedback in the EQUIPPED medication safety program. Acad Emerg Med. 2023;30(4):340-348. doi:10.1111/acem.14697 36790188

[53] Griffey RT, Lo HG, Burdick E, Keohane C, Bates DW. Guided medication dosing for elderly emergency patients using real-time, computerized decision support. J Am Med Inform Assoc. 2012;19(1):86-93. doi:10.1136/amiajnl-2011-000124 22052899 PMC3240752

[54] Kim M, Kaplan SJ, Mitchell SH, . The effect of computerized physician order entry template modifications on the administration of high-risk medications in older adults in the emergency department. Drugs Aging. 2017;34(10):793-801. doi:10.1007/s40266-017-0489-z 28956283

[55] Liu YL, Chu LL, Su HC, . Impact of computer-based and pharmacist-assisted medication review initiated in the emergency department. J Am Geriatr Soc. 2019;67(11):2298-2304. doi:10.1111/jgs.16078 31335969

[56] Terrell KM, Perkins AJ, Dexter PR, Hui SL, Callahan CM, Miller DK. Computerized decision support to reduce potentially inappropriate prescribing to older emergency department patients: a randomized, controlled trial. J Am Geriatr Soc. 2009;57(8):1388-1394. doi:10.1111/j.1532-5415.2009.02352.x 19549022

[57] Boyé ND, van der Velde N, de Vries OJ, ; IMPROveFALL trial collaborators. Effectiveness of medication withdrawal in older fallers: results from the Improving Medication Prescribing to Reduce Risk of Falls (IMPROveFALL) trial. Age Ageing. 2017;46(1):142-146. 28181639 10.1093/ageing/afw161

[58] Polinder S, Boyé ND, Mattace-Raso FU, ; IMPROveFALL trial collaborators. Cost-utility of medication withdrawal in older fallers: results from the Improving Medication Prescribing to Reduce Risk of Falls (IMPROveFALL) trial. BMC Geriatr. 2016;16(1):179. doi:10.1186/s12877-016-0354-7 27809792 PMC5096283

[59] Tan PJ, Khoo EM, Chinna K, . Individually-tailored multifactorial intervention to reduce falls in the Malaysian Falls Assessment and Intervention Trial (MyFAIT): a randomized controlled trial. PLoS One. 2018;13(8):e0199219. doi:10.1371/journal.pone.0199219 30074996 PMC6075745

[60] McGuinness LA, Higgins JPT. Risk-of-bias VISualization (robvis): An R package and Shiny web app for visualizing risk-of-bias assessments. Res Synth Methods. 2021;12(1):55-61. doi:10.1002/jrsm.1411 32336025

[61] Lin L, Chu H, Murad MH, . Empirical comparison of publication bias tests in meta-analysis. J Gen Intern Med. 2018;33(8):1260-1267. doi:10.1007/s11606-018-4425-7 29663281 PMC6082203

[62] Morgan SR, Acquisto NM, Coralic Z, . Clinical pharmacy services in the emergency department. Am J Emerg Med. 2018;36(10):1727-1732. doi:10.1016/j.ajem.2018.01.056 29475633

[63] Ortmann MJ, Johnson EG, Jarrell DH, . ASHP guidelines on emergency medicine pharmacist services. Am J Health Syst Pharm. 2021;78(3):261-275. doi:10.1093/ajhp/zxaa378 33480409

[64] Farmer BM, Hayes BD, Rao R, Farrell N, Nelson L. The role of clinical pharmacists in the emergency department. J Med Toxicol. 2018;14(1):114-116. doi:10.1007/s13181-017-0634-4 29075954 PMC6013729

[65] van Poelgeest EP, Seppala LJ, Lee JM, ; EuGMS SIG Pharmacology. Deprescribing practices, habits and attitudes of geriatricians and geriatricians-in-training across Europe: a large web-based survey. Eur Geriatr Med. 2022;13(6):1455-1466. doi:10.1007/s41999-022-00702-9 36319837 PMC9722796

[66] Reeve E, Thompson W, Farrell B. Deprescribing: a narrative review of the evidence and practical recommendations for recognizing opportunities and taking action. Eur J Intern Med. 2017;38:3-11. doi:10.1016/j.ejim.2016.12.021 28063660

[67] Stevens MB, Hastings SN, Powers J, . Enhancing the Quality of Prescribing Practices for Older Veterans Discharged from the Emergency Department (EQUiPPED): preliminary results from Enhancing Quality of Prescribing Practices for Older Veterans Discharged from the Emergency Department, a novel multicomponent interdisciplinary quality improvement initiative. J Am Geriatr Soc. 2015;63(5):1025-1029. doi:10.1111/jgs.13404 25945692 PMC13428995

[68] O’Brien MA, Rogers S, Jamtvedt G, . Educational outreach visits: effects on professional practice and health care outcomes. Cochrane Database Syst Rev. 2007;2007(4):CD000409. doi:10.1002/14651858.CD000409.pub2 17943742 PMC7032679

[69] Jankovic I, Chen JH. Clinical decision support and implications for the clinician burnout crisis. Yearb Med Inform. 2020;29(1):145-154. doi:10.1055/s-0040-1701986 32823308 PMC7442505

[70] Vandenberg AE, Vaughan CP, Stevens M, . Improving geriatric prescribing in the ED: a qualitative study of facilitators and barriers to clinical decision support tool use. Int J Qual Health Care. 2017;29(1):117-123. 27852639 10.1093/intqhc/mzw129

[71] Salwei ME, Hoonakker P, Carayon P, Wiegmann D, Pulia M, Patterson BW. Usability of a human factors-based clinical decision support in the emergency department: lessons learned for design and implementation. Hum Factors. 2024;66(3):647-657. doi:10.1177/00187208221078625 35420923 PMC9581441

[72] Muhiyaddin R, Elfadl A, Mohamed E, . Electronic health records and physician burnout: a scoping review. Stud Health Technol Inform. 2022;289:481-484. doi:10.3233/SHTI210962 35062195

[73] Khairat S, Coleman C, Ottmar P, Jayachander DI, Bice T, Carson SS. Association of electronic health record use with physician fatigue and efficiency. JAMA Netw Open. 2020;3(6):e207385. doi:10.1001/jamanetworkopen.2020.7385 32515799 PMC7284310

[74] Lee S, Cavalier FR, Hayes JM, . Delirium, confusion, or altered mental status as a risk for abnormal head CT in older adults in the emergency department: a systematic review and meta-analysis. Am J Emerg Med. 2023;71:190-194. doi:10.1016/j.ajem.2023.06.034 37423026

[75] Liu SW, Lee S, Hayes JM, ; Geriatric Emergency Department Delirium Guidelines Group. Head computed tomography findings in geriatric emergency department patients with delirium, altered mental status, and confusion: a systematic review. Acad Emerg Med. 2023;30(6):616-625. doi:10.1111/acem.14622 36330667

[76] Guyatt G, Oxman AD, Akl EA, . GRADE guidelines: 1. introduction—GRADE evidence profiles and summary of findings tables. J Clin Epidemiol. 2011;64(4):383-394. doi:10.1016/j.jclinepi.2010.04.026 21195583

[77] Fujita K, Hooper P, Masnoon N, . Impact of a comprehensive intervention bundle including the drug burden index on deprescribing anticholinergic and sedative drugs in older acute inpatients: a non-randomised controlled before-and-after pilot study. Drugs Aging. 2023;40(7):633-642. doi:10.1007/s40266-023-01032-6 37160561 PMC10299923

