# Supplementary material for: Emergency Department Programs to Support Medication Safety in Older Adults: A Systematic Review and Meta-Analysis
Source: JAMA Netw Open. 2025 Mar 11;8(3):e250814. doi: 10.1001/jamanetworkopen.2025.0814 (PMC11897843; doi:10.1001/jamanetworkopen.2025.0814)
Supplement: Supplement 1. — eMethods. Search Criteria and Strategies eFigure 1. Funnel Plots and Fail-Safe N Calculation Using Rosenberg Approach of Meta-Analyses eFigure 2. Results of Random-Effects Meta-Analysis Models eFigure 3. Extracted Data From Source eTable. Included Studies Inclusion and Exclusion Criteria [file jamanetwopen-e250814-s001.pdf]

Supplemental Online Content

Skains RM, Hayes JM, Selman K, et al; Geriatric Emergency Department Guidelines Medication Safety Group. Emergency department programs to support medication safety in older adults: a systematic review and meta-analysis. *JAMA Netw Open.* 2025;8(3):e250814. doi:10.1001/jamanetworkopen.2025.0814

eMethods. Search Criteria and Strategies

eFigure 1. Funnel Plots and Fail-Safe N Calculation Using Rosenberg Approach of Meta-Analyses

eFigure 2. Results of Random-Effects Meta-Analysis Models

eFigure 3. Extracted Data From Source

eTable. Included Studies Inclusion and Exclusion Criteria

This supplemental material has been provided by the authors to give readers additional information about their work.

© 2025 Skains RM et al. *JAMA Network Open*.

eMethods. Search Criteria and Strategies

Scopus – 1682 Results
TITLE-ABS ((deprescri* OR "de-prescri*" OR deintensif* OR "de-intensif*" OR polypharm* OR "poly-pharm*" OR "pill burden" OR "drug burden" OR "medication-related burden" OR "potentially inappropriate prescriptions" OR "inappropriate prescri*" OR "suboptimal prescri*" OR "sub-optimal prescri*" OR "unnecessary prescri*" OR "optimize prescri*" OR "optimizing prescri*" OR "optimise prescri*" OR "optimising prescri*" OR "prescribing pattern*" OR "ordering behav*" OR "potentially harmful prescri*" OR "over-prescri*" OR "overprescri*" OR "unnecessary treat*" OR "over-treat*" OR overtreat* OR "inappropriate medication*" OR pim OR pims OR "unnecessary medication*" OR "potentially harmful medication*" OR "inappropriate drug*" OR "unnecessary drug*" OR "potentially harmful drug*" OR "recommended dosing" OR "dosing recommendation*" OR "Prescribing Cascade*" OR "therapeutic duplicat*" OR "medication discontinuation" OR "discontinuing medication*" OR "drug discontinuation" OR "discontinuing drug*" OR ((adverse* OR "side effect*" OR toxicity OR inappropriate* OR unnecessary OR suboptimal OR reduc* OR stop* OR withdraw* OR ceas* OR discontinu* OR optimiz* OR optimis* OR overprescri* OR "over-prescri*") AND (pim OR pims OR pidp* OR medication* OR prescri* OR drug* OR polypharm*)))) AND TITLE-ABS (("medication review*" OR "medication therapy management" OR "medication management" OR "medication reconciliation" OR "pharmacist review*" OR "pharmacist intervention*" OR "pharmacist-led" OR "pharmacist involvement*" OR "medication optim*" OR "optimize medication*" OR "optimizing medication*" OR "optimise medication*" OR "optimising medication*" OR "guided dosing" OR "guided medication dosing" OR "guided prescription dosing" OR "guided drug dosing" OR "optimize drug*" OR "optimizing drug*" OR "optimise drug*" OR "optimising drug*" OR (review* AND ("drug utilization" OR "drug use")) OR "Order Entry System*" OR "computerized provider order entry" OR cpoe OR "decision support" OR "decision aid*" OR "decision rule*" OR "decision tool*" OR "academic detailing" OR "Medication Appropriateness" OR "beers criteria" OR "beers list" OR "beers protocol" OR stopp OR stoppfrail* OR "stopp-start" OR (equipped* AND (program* OR "prescribing practices" OR "medication safe*" OR "inappropriate medicat*")) OR "Screening Tool to Alert to Right Treatment" OR forta OR "Fit fOR The Aged" OR priscus OR norgep OR ((pim OR pims OR pidp* OR "potentially inappropriate" OR "medication review" OR prescri* OR inappropriate* OR overmedicat* OR overprescri* OR polypharm*) AND (avoid* OR reduc* OR quality OR improv* OR prevent* OR tool* OR strateg* OR protocol* OR pathway* OR program* OR review*)))) AND TITLE-ABS (("Emergency Medical Services" OR "Emergency Medical Technicians" OR "Emergency Treatment" OR "Emergency Medicine" OR "Emergency Department*" OR "trauma cent*" OR emergicenter* OR triag* OR "A&E" OR "pre-hospital" OR prehospital OR ems OR emt OR ambulance* OR ((emergency OR emergencies OR ems OR ed OR er) AND (service* OR dispatch* OR department* OR unit* OR ward* OR room* OR center* OR centre* OR system* OR visit* OR admiss* OR admit* OR consult*)) OR (trauma AND (support OR center OR centre OR department* OR unit* OR room*)))) AND TITLE-ABS (("age factors" OR geriatr* OR geront* OR elder* OR old OR older OR oldest OR eldest OR senior* OR frail* OR aged* OR aging OR agism OR "age factor*" OR "age-stratified" OR "age-related" OR "age-associated" OR "aging-related" OR retired OR retirement OR retiree* OR "social security" OR "nursing home*" OR "assisted living" OR pension* OR senium* OR senil* OR dementia OR grandparent* OR grandmother* OR grandfather* OR grandma* OR grandpa* OR sexagenarian* OR septuagenarian* OR octagenarian* OR nonagenarian* OR centenarian* OR supercentenarian*)) AND (LIMIT-TO (LANGUAGE , "English")) AND (EXCLUDE (DOCTYPE , "cp") OR EXCLUDE (DOCTYPE , "ch") OR EXCLUDE (DOCTYPE , "bk") OR EXCLUDE (DOCTYPE , "cr")) NOT (TITLE(pediatric* OR baby OR babies OR infan* OR child* OR boy OR boys OR girl OR girls OR "school-aged" OR juvenile* OR adolescen* OR youth) OR TITLE(mouse OR mice OR rat OR rats OR animal* OR murine))

Embase - 1176 Results
('deprescription'/exp OR 'polypharmacy'/exp OR 'prescribing error'/exp OR 'potentially inappropriate medication'/exp OR deprescri*:ti,ab,kw OR 'de-prescri*':ti,ab,kw OR deintensif*:ti,ab,kw OR 'de-intensif*':ti,ab,kw OR polypharm*:ti,ab,kw OR 'poly-pharm*':ti,ab,kw OR 'pill burden':ti,ab,kw OR 'drug burden':ti,ab,kw OR 'medication-related burden':ti,ab,kw OR 'inappropriate prescri*':ti,ab,kw OR 'suboptimal prescri*':ti,ab,kw OR 'sub-optimal prescri*':ti,ab,kw OR 'unnecessary prescri*':ti,ab,kw OR 'optimize prescri*':ti,ab,kw OR 'optimizing prescri*':ti,ab,kw OR 'optimise prescri*':ti,ab,kw OR 'optimising prescri*':ti,ab,kw OR 'prescribing

© 2025 Skains RM et al. *JAMA Network Open*.

pattern*':ti,ab,kw OR 'ordering behav*':ti,ab,kw OR 'potentially harmful prescri*':ti,ab,kw OR 'over-prescri*':ti,ab,kw OR 'overprescri*':ti,ab,kw OR 'unnecessary treat*':ti,ab,kw OR 'over-treat*':ti,ab,kw OR overtreat*:ti,ab,kw OR 'inappropriate medication*':ti,ab,kw OR pim:ti,ab,kw OR pims:ti,ab,kw OR 'unnecessary medication*':ti,ab,kw OR 'potentially harmful medication*':ti,ab,kw OR 'inappropriate drug*':ti,ab,kw OR 'unnecessary drug*':ti,ab,kw OR 'potentially harmful drug*':ti,ab,kw OR 'recommended dosing':ti,ab,kw OR 'dosing recommendation*':ti,ab,kw OR 'prescribing cascade*':ti,ab,kw OR 'therapeutic duplicat*':ti,ab,kw OR 'medication discontinuation':ti,ab,kw OR 'discontinuing medication*':ti,ab,kw OR 'drug discontinuation':ti,ab,kw OR 'discontinuing drug*':ti,ab,kw OR (('drug therapy'/exp OR 'drug mechanism'/exp OR 'prescription drug'/exp) AND ('adverse drug reaction'/lnk OR 'drug toxicity'/lnk OR 'side effect'/lnk)) OR 'adverse drug reaction'/exp OR ((adverse*:ti,ab,kw OR 'side effect*':ti,ab,kw OR toxicity:ti,ab,kw OR inappropriate*:ti OR unnecessary:ti OR suboptimal:ti OR reduc*:ti OR stop*:ti OR withdraw* OR ceas*:ti OR discontinu*:ti OR optimiz*:ti OR optimis*:ti OR overprescri*:ti OR 'over-prescri*':ti) AND (pim:ti,ab,kw OR pims:ti,ab,kw OR pidp*:ti,ab,kw OR medication*:ti,ab,kw OR prescri*:ti,ab,kw OR drug*:ti,ab,kw OR polypharm*:ti,ab,kw))) AND ("medication review*":ti,ab,kw OR "medication therapy management":ti,ab,kw OR "medication management":ti,ab,kw OR "medication reconciliation":ti,ab,kw OR "pharmacist review*":ti,ab,kw OR "pharmacist intervention*":ti,ab,kw OR "pharmacist-led":ti,ab,kw OR "pharmacist involvement*":ti,ab,kw OR "medication optim*":ti,ab,kw OR "optimize medication*":ti,ab,kw OR "optimizing medication*":ti,ab,kw OR "optimise medication*":ti,ab,kw OR "optimising medication*":ti,ab,kw OR "guided dosing":ti,ab,kw OR "guided medication dosing":ti,ab,kw OR "guided prescription dosing":ti,ab,kw OR "guided drug dosing":ti,ab,kw OR "optimize drug*":ti,ab,kw OR "optimizing drug*":ti,ab,kw OR "optimise drug*":ti,ab,kw OR "optimising drug*":ti,ab,kw OR (('prescribing error'/exp OR 'adverse drug reaction'/exp) AND 'prevention'/lnk) OR 'drug utilization review'/de OR 'medication therapy management'/exp OR (review*:ti,ab,kw AND ("drug utilization":ti,ab,kw OR "drug use":ti,ab,kw)) OR 'clinical decision support system'/exp OR 'physician order entry system'/exp OR "computerized provider order entry":ti,ab,kw OR CPOE:ti,ab,kw OR "decision support":ti,ab,kw OR "decision aid*":ti,ab,kw OR "decision rule*":ti,ab,kw OR "decision tool*":ti,ab,kw OR “academic detailing”:ti,ab,kw OR "Medication Appropriateness":ti,ab,kw OR "beers criteria":ti,ab,kw OR "beers list":ti,ab,kw OR "beers protocol":ti,ab,kw OR stopp:ti,ab,kw OR stoppfrail*:ti,ab,kw OR "stopp-start":ti,ab,kw OR (equipped*:ti,ab,kw AND (program*:ti,ab,kw OR "prescribing practices":ti,ab,kw OR "medication safe*":ti,ab,kw OR "inappropriate medicat*":ti,ab,kw)) OR "Screening Tool to Alert to Right Treatment":ti,ab,kw OR FORTA:ti,ab,kw OR "Fit fOR The Aged":ti,ab,kw OR PRISCUS:ti,ab,kw OR NORGEP:ti,ab,kw OR ((PIM:ti,ab,kw OR PIMS:ti,ab,kw OR PIDP*:ti,ab,kw OR "potentially inappropriate":ti,ab,kw OR "medication review":ti OR prescri*:ti OR inappropriate*:ti OR overmedicat*:ti OR overprescri*:ti OR polypharm*:ti) AND (avoid*:ti OR reduc*:ti OR quality:ti OR improv*:ti OR prevent*:ti OR tool*:ti OR strateg*:ti OR protocol*:ti OR pathway*:ti OR program*:ti OR review*:ti))) AND ('hospital emergency service'/exp OR 'emergency medicine'/exp OR 'emergency'/exp OR 'emergency health service'/exp OR 'rescue personnel'/exp OR 'emergency treatment'/exp OR 'ambulance'/exp OR "Emergency Medicine":ti,ab,kw OR "Emergency Department*":ti,ab,kw OR "trauma cent*":ti,ab,kw OR emergicenter*:ti,ab,kw OR triag*:ti,ab,kw OR 'a&e':ti,ab,kw OR 'pre-hospital':ti,ab,kw OR prehospital:ti,ab,kw OR ems:ti,ab,kw OR emt:ti,ab,kw OR ambulance*:ti,ab,kw OR ((emergency:ti,ab,kw OR emergencies:ti,ab,kw OR ems:ti,ab,kw OR ed:ti,ab,kw OR er:ti,ab,kw) AND (service*:ti,ab,kw OR dispatch*:ti,ab,kw OR department*:ti,ab,kw OR unit*:ti,ab,kw OR ward*:ti,ab,kw OR room*:ti,ab,kw OR center*:ti,ab,kw OR centre*:ti,ab,kw OR system*:ti,ab,kw OR visit*:ti,ab,kw OR admiss*:ti,ab,kw OR admit*:ti,ab,kw OR consult*:ti,ab,kw)) OR (trauma:ti,ab,kw AND (support:ti,ab,kw OR center:ti,ab,kw OR centre:ti,ab,kw OR department*:ti,ab,kw OR unit*:ti,ab,kw OR room*:ti,ab,kw)) OR emergen*:jt) AND ('aged'/exp OR 'elderly care'/exp OR 'geriatric assessment'/exp OR 'geriatrics'/exp OR 'gerontopsychiatry'/exp OR 'geriatric nursing'/exp OR 'geriatric dentistry'/exp OR 'nursing home'/exp OR 'home for the aged'/exp OR 'age'/exp OR geriatr*:ti,ab,kw OR geront*:ti,ab,kw OR elder*:ti,ab,kw OR old:ti,ab,kw OR older:ti,ab,kw OR oldest:ti,ab,kw OR eldest:ti,ab,kw OR senior*:ti,ab,kw OR frail*:ti,ab,kw OR aged*:ti,ab,kw OR aging:ti,ab,kw OR agism:ti,ab,kw OR 'age factor*':ti,ab,kw OR 'age-stratified':ti,ab,kw OR 'age-related':ti,ab,kw OR 'age-associated':ti,ab,kw OR 'aging-related':ti,ab,kw OR retired:ti,ab,kw OR retirement:ti,ab,kw OR retiree*:ti,ab,kw OR 'social security':ti,ab,kw OR 'nursing home*':ti,ab,kw OR 'assisted living':ti,ab,kw OR pension*:ti,ab,kw OR senium*:ti,ab,kw OR senil*:ti,ab,kw OR dementia:ti,ab,kw OR grandparent*:ti,ab,kw OR grandmother*:ti,ab,kw OR grandfather*:ti,ab,kw OR grandma*:ti,ab,kw OR grandpa*:ti,ab,kw OR sexagenarian*:ti,ab,kw OR septuagenarian*:ti,ab,kw OR octagenarian*:ti,ab,kw OR nonagenarian*:ti,ab,kw OR centenarian*:ti,ab,kw OR supercentenarian*:ti,ab,kw) AND [english]/lim NOT (([newborn]/lim OR [infant]/lim OR [child]/lim OR [adolescent]/lim) NOT ([adult]/lim OR [aged]/lim OR [very elderly]/lim) OR pediatric*:ti OR baby:ti OR babies:ti OR infan*:ti OR child*:ti OR boy:ti OR boys:ti OR girl:ti OR girls:ti OR 'school-aged':ti OR juvenile*:ti OR adolescen*:ti OR youth:ti) NOT ([animals]/lim NOT

© 2025 Skains RM et al. *JAMA Network Open*.

[humans]/lim OR mouse:ti OR mice:ti OR rat:ti OR rats:ti OR animal*:ti OR murine:ti) NOT ([conference abstract]/lim OR [conference paper]/lim OR [conference review]/lim)

PubMed – 724 Results
("Deprescriptions"[mesh] OR "Polypharmacy"[mesh] OR "Inappropriate Prescribing"[mesh] OR "Potentially Inappropriate Medication List"[mesh] OR deprescri*[tw] OR "de-prescri*"[tw] OR deintensif*[tw] OR "de-intensif*"[tw] OR polypharm*[tw] OR "poly-pharm*"[tw] OR "pill burden"[tw] OR "drug burden"[tw] OR "medication-related burden"[tw] OR "inappropriate prescri*"[tw] OR "suboptimal prescri*"[tw] OR "sub-optimal prescri*"[tw] OR "unnecessary prescri*"[tw] OR "optimize prescri*"[tw] OR "optimizing prescri*"[tw] OR "optimise prescri*"[tw] OR "optimising prescri*"[tw] OR "prescribing pattern*"[tw] OR "ordering behav*"[tw] OR "potentially harmful prescri*"[tw] OR "over-prescri*"[tw] OR "overprescri*"[tw] OR "unnecessary treat*"[tw] OR "over-treat*"[tw] OR overtreat*[tw] OR "inappropriate medication*"[tw] OR PIM[tw] OR PIMS[tw] OR "unnecessary medication*"[tw] OR "potentially harmful medication*"[tw] OR "inappropriate drug*"[tw] OR "unnecessary drug*"[tw] OR "potentially harmful drug*"[tw] OR "recommended dosing"[tw] OR "dosing recommendation*"[tw] OR "Prescribing Cascade*"[tw] OR "therapeutic duplicat*"[tw] OR "medication discontinuation"[tw] OR "discontinuing medication*"[tw] OR "drug discontinuation"[tw] OR "discontinuing drug*"[tw] OR "Drug Therapy/adverse effects"[Mesh] OR "Pharmacologic Actions/adverse effects"[Mesh] OR "Prescription Drugs/adverse effects"[Mesh] OR "Drug-Related Side Effects and Adverse Reactions"[Mesh] OR ((adverse*[tw] OR "side effect*"[tw] OR toxicity[tw] OR inappropriate*[ti] OR unnecessary[ti] OR suboptimal[ti] OR reduc*[ti] OR stop*[ti] OR withdraw* OR ceas*[ti] OR discontinu*[ti] OR optimiz*[ti] OR optimis*[ti] OR overprescri*[ti] OR "over-prescri*"[ti]) AND (PIM[tw] OR PIMS[tw] OR PIDP*[tw] OR medication*[tw] OR prescri*[tw] OR drug*[tw] OR polypharm*[tw]))) AND ("medication review*"[tw] OR "medication optim*"[tw] OR "optimize medication*"[tw] OR "optimizing medication*"[tw] OR "optimise medication*"[tw] OR "optimising medication*"[tw] OR "guided dosing"[tw] OR "guided medication dosing"[tw] OR "guided prescription dosing"[tw] OR "guided drug dosing"[tw] OR "optimize drug*"[tw] OR "optimizing drug*"[tw] OR "optimise drug*"[tw] OR "optimising drug*"[tw] OR "Inappropriate Prescribing/prevention and control"[MeSH] OR "Drug-Related Side Effects and Adverse Reactions/prevention and control"[MeSH] OR "Drug Utilization Review"[Majr:NoExp] OR (review*[tw] AND ("drug utilization"[tw] OR "drug use"[tw])) OR "Decision Support Systems, Clinical"[Mesh] OR "Medical Order Entry Systems"[Mesh] OR "computerized provider order entry"[tw] OR CPOE[tw] OR "decision support"[tw] OR "decision aid*"[tw] OR "decision rule*"[tw] OR "decision tool*"[tw] OR “academic detailing”[tw] OR "Medication Appropriateness"[tw] OR "beers criteria"[tw] OR "beers list"[tw] OR "beers protocol"[tw] OR stopp[tw] OR stoppfrail*[tw] OR "stopp start"[tw] OR (equipped*[tw] AND (program*[tw] OR "prescribing practices"[tw] OR "medication safe*"[tw] OR "inappropriate medicat*"[tw])) OR "Screening Tool to Alert to Right Treatment"[tw] OR FORTA[tw] OR "Fit fOR The Aged"[tw] OR PRISCUS[tw] OR NORGEP[tw] OR ((PIM[tw] OR PIMS[tw] OR PIDP*[tw] OR "potentially inappropriate"[tw] OR prescri*[ti] OR inappropriate*[ti] OR overmedicat*[ti] OR overprescri*[ti] OR polypharm*[ti]) AND (avoid*[ti] OR reduc*[ti] OR quality[ti] OR improv*[ti] OR prevent*[ti] OR tool*[ti] OR strateg*[ti] OR protocol*[ti] OR pathway*[ti] OR program*[ti] OR review*[ti]))) AND ("Emergency Service, Hospital"[Mesh] OR "Emergency Medicine"[Mesh] OR "Emergencies"[Mesh] OR "Emergency Medical Services"[MeSH] OR "Emergency Medical Technicians"[Mesh] OR "Emergency Treatment"[Mesh] OR "Ambulances"[Mesh] OR "Emergency Medicine"[tw] OR "Emergency Department*"[tw] OR "trauma cent*"[tw] OR emergicenter*[tw] OR triag*[tw] OR ED[tw] OR ER[tw] OR "emergency"[tw] OR "emergencies"[tw] OR "A&E"[tw] OR "pre-hospital"[tw] OR prehospital[tw] OR EMS[tw] OR EMT[tw] OR ambulance*[tw] OR ((Emergency[tw] OR emergencies[tw] OR EMS[tw] OR ED[tw] OR ER[tw]) AND (service*[tw] OR dispatch*[tw] OR department*[tw] OR unit*[tw] OR ward*[tw] OR room*[tw] OR center*[tw] OR centre*[tw] OR system*[tw] OR visit*[tw] OR admiss*[tw] OR admit*[tw] OR consult*[tw])) OR (trauma[tw] AND (support[tw] OR center[tw] OR centre[tw] OR department*[tw] OR unit*[tw] OR room*[tw])) OR emergen*[so]) AND ("Aged"[MeSH] OR "Health Services for the Aged"[mesh] OR "Geriatric Assessment"[mesh] OR "Geriatrics"[mesh] OR "Geriatric Psychiatry"[mesh] OR "Geriatric Nursing"[mesh] OR "Geriatric Dentistry"[mesh] OR "Dental Care for Aged"[mesh] OR "Homes for the Aged"[mesh] OR "Nursing Homes"[mesh] OR "Housing for the Elderly"[mesh] OR "age factors"[mesh] OR geriatr*[tw] OR geront*[tw] OR elder*[tw] OR old[tw] OR older[tw] OR oldest[tw] OR eldest[tw] OR senior*[tw] OR aged*[tw] OR aging[tw] OR agism[tw] OR "age factor*"[tw] OR "age-stratified"[tw] OR "age-related"[tw] OR "age-associated"[tw] OR "aging-related"[tw] OR retired[tw] OR retirement[tw] OR Retiree*[tw] OR "social security"[tw] OR "nursing home*"[tw] OR "assisted living"[tw] OR pension*[tw] OR senium*[tw] OR senil*[tw] OR dementia[tw] OR grandparent*[tw] OR grandmother*[tw] OR grandfather*[tw] OR grandma*[tw] OR grandpa*[tw] OR sexagenarian*[tw] OR septuagenarian*[tw] OR octagenarian*[tw] OR nonagenarian*[tw] OR centenarian*[tw] OR supercentenarian*[tw])

© 2025 Skains RM et al. *JAMA Network Open*.

AND English[Filter] NOT ((allchild[Filter] NOT alladult[Filter]) OR pediatric*[ti] OR baby[ti] OR babies[ti] OR infan*[ti] OR child*[ti] OR boy[ti] OR boys[ti] OR girl[ti] OR girls[ti] OR "school-aged"[ti] OR juvenile*[ti] OR adolescen*[ti] OR youth[ti]) NOT ((animal[Filter] NOT humans[Filter]) OR mouse[ti] OR mice[ti] OR rat[ti] OR rats[ti] OR animal*[ti] OR murine[ti])

PsycInfo - 603 Results
(DE "Prescribing (Drugs)" OR DE "Polypharmacy" OR deprescri* OR "de-prescri*" OR deintensif* OR "de-intensif*" OR polypharm* OR "poly-pharm*" OR "pill burden" OR "drug burden" OR "medication-related burden" OR "inappropriate prescri*" OR "suboptimal prescri*" OR "sub-optimal prescri*" OR "unnecessary prescri*" OR "optimize prescri*" OR "optimizing prescri*" OR "optimise prescri*" OR "optimising prescri*" OR "prescribing pattern*" OR "ordering behav*" OR "potentially harmful prescri*" OR "over-prescri*" OR "overprescri*" OR "unnecessary treat*" OR "over-treat*" OR overtreat* OR "inappropriate medication*" OR PIM OR PIMS OR "unnecessary medication*" OR "potentially harmful medication*" OR "inappropriate drug*" OR "unnecessary drug*" OR "potentially harmful drug*" OR "recommended dosing" OR "dosing recommendation*" OR "Prescribing Cascade*" OR "therapeutic duplicat*" OR "medication discontinuation" OR "discontinuing medication*" OR "drug discontinuation" OR "discontinuing drug*" OR ((adverse* OR "side effect*" OR toxicity OR TI(inappropriate* OR unnecessary OR suboptimal OR reduc* OR stop* OR withdraw* OR ceas* OR discontinu* OR optimiz* OR optimis* OR overprescri* OR "over-prescri*")) AND (PIM OR PIMS OR PIDP* OR medication* OR prescri* OR drug* OR polypharm*))) AND ("medication review*" OR "medication optim*" OR "optimize medication*" OR "optimizing medication*" OR "optimise medication*" OR "optimising medication*" OR "guided dosing" OR "guided medication dosing" OR "guided prescription dosing" OR "guided drug dosing" OR "optimize drug*" OR "optimizing drug*" OR "optimise drug*" OR "optimising drug*" OR (review* AND ("drug utilization" OR "drug use")) OR "computerized provider order entry" OR CPOE OR DE "Decision Support Systems" OR "decision support" OR "decision aid*" OR "decision rule*" OR "decision tool*" OR “academic detailing” OR "Medication Appropriateness" OR "beers criteria" OR "beers list" OR "beers protocol" OR stopp OR stoppfrail* OR "stopp start" OR (equipped* AND (program* OR "prescribing practices" OR "medication safe*" OR "inappropriate medicat*")) OR "Screening Tool to Alert to Right Treatment" OR FORTA OR "Fit fOR The Aged" OR PRISCUS OR NORGEP OR ((PIM OR PIMS OR PIDP* OR "potentially inappropriate" OR TI(prescri* OR inappropriate* OR overmedicat* OR overprescri* OR polypharm*)) AND TI(avoid* OR reduc* OR quality OR improv* OR prevent* OR tool* OR strateg* OR protocol* OR pathway* OR program* OR review*)))) AND (DE "Emergency Services" OR "Emergency Medicine" OR "Emergency Department*" OR "trauma cent*" OR emergicenter* OR triag* OR "ED" OR "ER" OR "emergency" OR "emergencies" OR "A&E" OR "pre-hospital" OR prehospital OR EMS OR EMT OR ambulance* OR ((emergency OR emergencies OR ems OR ed OR er) AND (service* OR dispatch* OR department* OR unit* OR ward* OR room* OR center* OR centre* OR system* OR visit* OR admiss* OR admit* OR consult*)) OR (trauma AND (support OR center OR centre OR department* OR unit* OR room*))) OR SO "emergen*") AND (DE "Aging" OR DE "Geriatric Assessment" OR DE "Geriatric Patients" OR DE "Older Adulthood" OR DE "Elder Care" OR DE "Geratric Psychiatry" OR DE "Nursing Homes" OR AG (Aged (65 yrs & older) OR Very Old (85 yrs & older)) OR geriatr* OR geront* OR elder* OR old OR older OR oldest OR eldest OR senior* OR aged* OR aging OR agism OR "age factor*" OR "age-stratified" OR "age-related" OR "age-associated" OR "aging-related" OR retired OR retirement OR Retiree* OR "social security" OR "nursing home*" OR "assisted living" OR pension* OR senium* OR senil* OR dementia OR grandparent* OR grandmother* OR grandfather* OR grandma* OR grandpa* OR sexagenarian* OR septuagenarian* OR octagenarian* OR nonagenarian* OR centenarian* OR supercentenarian*) AND LA English NOT (TI(pediatric* OR baby OR babies OR infan* OR child* OR boy OR boys OR girl OR girls OR "school-aged" OR juvenile* OR adolescen* OR youth) OR TI(mouse OR mice OR rat OR rats OR animal* OR murine))
Plus filters:
AND Academic Journals
NOT clinical trials

ProQuest Central – 462 Results
(((ti((inappropriate* OR unnecessary OR suboptimal OR reduc* OR stop* OR withdraw* OR ceas* OR discontinu* OR optimiz* OR optimis* OR overprescri* OR "over-prescri*")) AND (PIM OR PIMS OR PIDP* OR medication* OR prescri* OR drug* OR polypharm*)) OR (noft(adverse* OR ("side effect" OR "side effects") OR toxicity) AND (PIM OR PIMS OR PIDP* OR medication* OR prescri* OR drug* OR polypharm*)) OR noft(deprescri* OR "de-prescri*" OR deintensif* OR "de-intensif*" OR polypharm* OR "poly-pharm*" OR "pill burden" OR "drug burden" OR "medication-related burden" OR "inappropriate prescri*" OR "suboptimal prescri*" OR "sub-optimal prescri*"

OR "unnecessary prescri*" OR "optimize prescri*" OR "optimizing prescri*" OR "optimise prescri*" OR "optimising prescri*" OR "prescribing pattern*" OR "ordering behav*" OR "potentially harmful prescri*" OR "over-prescri*" OR "overprescri*" OR "unnecessary treat*" OR "over-treat*" OR overtreat* OR "inappropriate medication*" OR PIM OR PIMS OR "unnecessary medication*" OR "potentially harmful medication*" OR "inappropriate drug*" OR "unnecessary drug*" OR "potentially harmful drug*" OR "recommended dosing" OR "dosing recommendation*" OR "Prescribing Cascade*" OR "therapeutic duplicat*" OR "medication discontinuation" OR "discontinuing medication*" OR "drug discontinuation" OR "discontinuing drug*")) AND (noft("medication review*" OR "medication optim*" OR "optimize medication*" OR "optimizing medication*" OR "optimise medication*" OR "optimising medication*" OR "guided dosing" OR "guided medication dosing" OR "guided prescription dosing" OR "guided drug dosing" OR "optimize drug*" OR "optimizing drug*" OR "optimise drug*" OR "optimising drug*" OR (review* AND ("drug utilization" OR "drug use")) OR "computerized provider order entry" OR CPOE OR "decision support" OR "decision aid*" OR "decision rule*" OR "decision tool*" OR “academic detailing” OR "Medication Appropriateness" OR "beers criteria" OR "beers list" OR "beers protocol" OR stopp OR stoppfrail* OR "stopp start" OR (equipped* AND (program* OR "prescribing practices" OR "medication safe*" OR "inappropriate medicat*")) OR "Screening Tool to Alert to Right Treatment" OR FORTA OR "Fit fOR The Aged" OR PRISCUS OR NORGEP) OR (ti((avoid* OR reduc* OR quality OR improv* OR prevent* OR tool* OR strateg* OR protocol* OR pathway* OR program* OR review*)) AND (PIM OR PIMS OR PIDP* OR "potentially inappropriate")) OR ti((prescri* OR inappropriate* OR overmedicat* OR overprescri* OR polypharm*) AND (avoid* OR reduc* OR quality OR improv* OR prevent* OR tool* OR strateg* OR protocol* OR pathway* OR program* OR review*))) AND noft("Emergency Medicine" OR "Emergency Department*" OR "trauma cent*" OR emergicenter* OR triag* OR "ED" OR "ER" OR "emergency" OR "emergencies" OR "A&E" OR "pre-hospital" OR prehospital OR EMS OR EMT OR ambulance* OR ((emergency OR emergencies OR ems OR ed OR er) AND (service* OR dispatch* OR department* OR unit* OR ward* OR room* OR center* OR centre* OR system* OR visit* OR admiss* OR admit* OR consult*)) OR (trauma AND (support OR center OR centre OR department* OR unit* OR room*))) AND noft(geriatr* OR geront* OR elder* OR old OR older OR oldest OR eldest OR senior* OR aged* OR aging OR agism OR "age factor*" OR "age-stratified" OR "age-related" OR "age-associated" OR "aging-related" OR retired OR retirement OR Retiree* OR "social security" OR "nursing home*" OR "assisted living" OR pension* OR senium* OR senil* OR dementia OR grandparent* OR grandmother* OR grandfather* OR grandma* OR grandpa* OR sexagenarian* OR septuagenarian* OR octagenarian* OR nonagenarian* OR centenarian* OR supercentenarian*)) NOT ti((pediatric* OR baby OR babies OR infan* OR child* OR boy OR boys OR girl OR girls OR "school-aged" OR juvenile* OR adolescen* OR youth) OR (mouse OR mice OR rat OR rats OR animal* OR murine))

CINAHL – 358 Results
(MH "Deprescribing" OR MH "Polypharmacy" OR MH "Inappropriate Prescribing" OR "Potentially Inappropriate Medication List"[mesh] OR deprescri* OR "de-prescri*" OR deintensif* OR "de-intensif*" OR polypharm* OR "poly-pharm*" OR "pill burden" OR "drug burden" OR "medication-related burden" OR "inappropriate prescri*" OR "suboptimal prescri*" OR "sub-optimal prescri*" OR "unnecessary prescri*" OR "optimize prescri*" OR "optimizing prescri*" OR "optimise prescri*" OR "optimising prescri*" OR "prescribing pattern*" OR "ordering behav*" OR "potentially harmful prescri*" OR "over-prescri*" OR "overprescri*" OR "unnecessary treat*" OR "over-treat*" OR overtreat* OR "inappropriate medication*" OR PIM OR PIMS OR "unnecessary medication*" OR "potentially harmful medication*" OR "inappropriate drug*" OR "unnecessary drug*" OR "potentially harmful drug*" OR "recommended dosing" OR "dosing recommendation*" OR "Prescribing Cascade*" OR "therapeutic duplicat*" OR "medication discontinuation" OR "discontinuing medication*" OR "drug discontinuation" OR "discontinuing drug*" OR MH "Drug Therapy+/AE" OR MH "Drugs, Prescription+/AE" OR MH "Adverse Drug Event+" OR ((adverse* OR "side effect*" OR toxicity OR TI(inappropriate* OR unnecessary OR suboptimal OR reduc* OR stop* OR withdraw* OR ceas* OR discontinu* OR optimiz* OR optimis* OR overprescri* OR "over-prescri*")) AND (PIM OR PIMS OR PIDP* OR medication* OR prescri* OR drug* OR polypharm*))) AND (MH "Medication Management" OR MH "Medication Reconciliation" OR "medication review*" OR "medication optim*" OR "optimize medication*" OR "optimizing medication*" OR "optimise medication*" OR "optimising medication*" OR "guided dosing" OR "guided medication dosing" OR "guided prescription dosing" OR "guided drug dosing" OR "optimize drug*" OR "optimizing drug*" OR "optimise drug*" OR "optimising drug*" OR (review* AND (MH "Drug Utilization" OR "drug utilization" OR "drug use")) OR MH "Decision Support Systems, Clinical" OR MH "Electronic Order Entry" OR "computerized provider order entry" OR CPOE OR "decision support" OR "decision aid*" OR "decision rule*" OR "decision tool*" OR “academic detailing” OR "Medication Appropriateness" OR "beers criteria" OR "beers list" OR "beers protocol" OR stopp OR stoppfrail* OR "stopp start"

© 2025 Skains RM et al. *JAMA Network Open*.

OR (equipped* AND (program* OR "prescribing practices" OR "medication safe*" OR "inappropriate medicat*")) OR "Screening Tool to Alert to Right Treatment" OR FORTA OR "Fit fOR The Aged" OR PRISCUS OR NORGEP OR ((PIM OR PIMS OR PIDP* OR "potentially inappropriate" OR TI(prescri* OR inappropriate* OR overmedicat* OR overprescri* OR polypharm*)) AND TI(avoid* OR reduc* OR quality OR improv* OR prevent* OR tool* OR strateg* OR protocol* OR pathway* OR program* OR review*)))) AND (MH "Emergencies+" OR MH "Emergency Service" OR MH "Emergency Medical Services" OR MH "Emergency Services, Psychiatric" OR MH "Psychiatric Emergencies" OR MH "Emergency Patients" OR MH "Physicians, Emergency" OR MH "Emergency Nurse Practitioners" OR MH "Emergency Nursing" OR MH "Emergency Medicine" OR MH "Emergency Care" OR MH "Emergency Medical Technicians" OR MH "Prehospital Care" OR MH "Ambulances" OR MH "Trauma Centers" OR "Emergency Medicine" OR "Emergency Department*" OR "trauma cent*" OR emergicenter* OR triag* OR "ED" OR "ER" OR "emergency" OR "emergencies" OR "A&E" OR "pre-hospital" OR prehospital OR EMS OR EMT OR ambulance* OR ((emergency OR emergencies OR ems OR ed OR er) AND (service* OR dispatch* OR department* OR unit* OR ward* OR room* OR center* OR centre* OR system* OR visit* OR admiss* OR admit* OR consult*)) OR (trauma AND (support OR center OR centre OR department* OR unit* OR room*))) OR SO "emergen*") AND (MH "Age Specific Care" OR MH "Age Factors" OR MH "Ageism" OR MH "Aged+" OR MH "Hospitalization of Older Persons" OR MH "Health Services for Older Persons" OR MH "Dental Care for Older Persons" OR MH "Rehabilitation, Geriatric" OR MH "Housing for Older Persons" OR MH "Gerontologic Nursing+" OR MH "Gerontologic Care" OR MH "Assisted Living" OR MH "Aging+" OR geriatr* OR geront* OR elder* OR old OR older OR oldest OR eldest OR senior* OR aged* OR aging OR agism OR "age factor*" OR "age-stratified" OR "age-related" OR "age-associated" OR "aging-related" OR retired OR retirement OR Retiree* OR "social security" OR "nursing home*" OR "assisted living" OR pension* OR senium* OR senil* OR dementia OR grandparent* OR grandmother* OR grandfather* OR grandma* OR grandpa* OR sexagenarian* OR septuagenarian* OR octagenarian* OR nonagenarian* OR centenarian* OR supercentenarian*) AND LA English NOT (((MH "Child+" OR MH "Adolescence+" OR MH "Minors (Legal)") NOT MH "Adult+") OR TI(pediatric* OR baby OR babies OR infan* OR child* OR boy OR boys OR girl OR girls OR "school-aged" OR juvenile* OR adolescen* OR youth)) NOT ((MH "Animals+" NOT MH "Human") OR TI(mouse OR mice OR rat OR rats OR animal* OR murine))

AgeLine - 161 results
(deprescri* OR "de-prescri*" OR deintensif* OR "de-intensif*" OR polypharm* OR "poly-pharm*" OR "pill burden" OR "drug burden" OR "medication-related burden" OR "inappropriate prescri*" OR "suboptimal prescri*" OR "sub-optimal prescri*" OR "unnecessary prescri*" OR "optimize prescri*" OR "optimizing prescri*" OR "optimise prescri*" OR "optimising prescri*" OR "prescribing pattern*" OR "ordering behav*" OR "potentially harmful prescri*" OR "over-prescri*" OR "overprescri*" OR "unnecessary treat*" OR "over-treat*" OR overtreat* OR "inappropriate medication*" OR PIM OR PIMS OR "unnecessary medication*" OR "potentially harmful medication*" OR "inappropriate drug*" OR "unnecessary drug*" OR "potentially harmful drug*" OR "recommended dosing" OR "dosing recommendation*" OR "Prescribing Cascade*" OR "therapeutic duplicat*" OR "medication discontinuation" OR "discontinuing medication*" OR "drug discontinuation" OR "discontinuing drug*" OR ((adverse* OR "side effect*" OR toxicity OR TI(inappropriate* OR unnecessary OR suboptimal OR reduc* OR stop* OR withdraw* OR ceas* OR discontinu* OR optimiz* OR optimis* OR overprescri* OR "over-prescri*")) AND (PIM OR PIMS OR PIDP* OR medication* OR prescri* OR drug* OR polypharm*))) AND ("medication review*" OR "medication optim*" OR "optimize medication*" OR "optimizing medication*" OR "optimise medication*" OR "optimising medication*" OR "guided dosing" OR "guided medication dosing" OR "guided prescription dosing" OR "guided drug dosing" OR "optimize drug*" OR "optimizing drug*" OR "optimise drug*" OR "optimising drug*" OR (review* AND ("drug utilization" OR "drug use")) OR "computerized provider order entry" OR CPOE OR "decision support" OR "decision aid*" OR "decision rule*" OR "decision tool*" OR “academic detailing” OR "Medication Appropriateness" OR "beers criteria" OR "beers list" OR "beers protocol" OR stopp OR stoppfrail* OR "stopp start" OR (equipped* AND (program* OR "prescribing practices" OR "medication safe*" OR "inappropriate medicat*")) OR "Screening Tool to Alert to Right Treatment" OR FORTA OR "Fit fOR The Aged" OR PRISCUS OR NORGEP OR ((PIM OR PIMS OR PIDP* OR "potentially inappropriate" OR TI(prescri* OR inappropriate* OR overmedicat* OR overprescri* OR polypharm*)) AND TI(avoid* OR reduc* OR quality OR improv* OR prevent* OR tool* OR strateg* OR protocol* OR pathway* OR program* OR review*)))) AND ("Emergency Medicine" OR "Emergency Department*" OR "trauma cent*" OR emergicenter* OR triag* OR "ED" OR "ER" OR "emergency" OR "emergencies" OR "A&E" OR "pre-hospital" OR prehospital OR EMS OR EMT OR ambulance* OR ((emergency OR emergencies OR ems OR ed OR er) AND (service* OR dispatch* OR department* OR unit* OR ward* OR room* OR center* OR centre* OR system* OR visit*

© 2025 Skains RM et al. *JAMA Network Open*.

OR admiss* OR admit* OR consult*)) OR (trauma AND (support OR center OR centre OR department* OR unit* OR room*))) OR SO "emergen*") AND (geriatr* OR geront* OR elder* OR old OR older OR oldest OR eldest OR senior* OR aged* OR aging OR agism OR "age factor*" OR "age-stratified" OR "age-related" OR "age-associated" OR "aging-related" OR retired OR retirement OR Retiree* OR "social security" OR "nursing home*" OR "assisted living" OR pension* OR senium* OR senil* OR dementia OR grandparent* OR grandmother* OR grandfather* OR grandma* OR grandpa* OR sexagenarian* OR septuagenarian* OR octagenarian* OR nonagenarian* OR centenarian* OR supercentenarian*) AND LA English NOT (TI(pediatric* OR baby OR babies OR infan* OR child* OR boy OR boys OR girl OR girls OR "school-aged" OR juvenile* OR adolescen* OR youth) OR TI(mouse OR mice OR rat OR rats OR animal* OR murine))

Cochrane Library – 30 Results
((deprescri* OR "de-prescri*" OR deintensif* OR "de-intensif*" OR polypharm* OR "poly-pharm*" OR "pill burden" OR "drug burden" OR "medication-related burden" OR "inappropriate prescri*" OR "suboptimal prescri*" OR "sub-optimal prescri*" OR "unnecessary prescri*" OR "optimize prescri*" OR "optimizing prescri*" OR "optimise prescri*" OR "optimising prescri*" OR "prescribing pattern*" OR "ordering behav*" OR "potentially harmful prescri*" OR "over-prescri*" OR "overprescri*" OR "unnecessary treat*" OR "over-treat*" OR overtreat* OR "inappropriate medication*" OR PIM OR PIMS OR "unnecessary medication*" OR "potentially harmful medication*" OR "inappropriate drug*" OR "unnecessary drug*" OR "potentially harmful drug*" OR "recommended dosing" OR "dosing recommendation*" OR "Prescribing Cascade*" OR "therapeutic duplicat*" OR "medication discontinuation" OR "discontinuing medication*" OR "drug discontinuation" OR "discontinuing drug*" OR ((adverse* OR "side effect*" OR toxicity OR TI(inappropriate* OR unnecessary OR suboptimal OR reduc* OR stop* OR withdraw* OR ceas* OR discontinu* OR optimiz* OR optimis* OR overprescri* OR "over-prescri*")) AND (PIM OR PIMS OR PIDP* OR medication* OR prescri* OR drug* OR polypharm*)))):ti,ab,kw AND ("medication review*" OR "medication optim*" OR "optimize medication*" OR "optimizing medication*" OR "optimise medication*" OR "optimising medication*" OR "guided dosing" OR "guided medication dosing" OR "guided prescription dosing" OR "guided drug dosing" OR "optimize drug*" OR "optimizing drug*" OR "optimise drug*" OR "optimising drug*" OR (review* AND ("drug utilization" OR "drug use")) OR "computerized provider order entry" OR CPOE OR "decision support" OR "decision aid*" OR "decision rule*" OR "decision tool*" OR “academic detailing” OR "Medication Appropriateness" OR "beers criteria" OR "beers list" OR "beers protocol" OR stopp OR stoppfrail* OR "stopp start" OR (equipped* AND (program* OR "prescribing practices" OR "medication safe*" OR "inappropriate medicat*")) OR "Screening Tool to Alert to Right Treatment" OR FORTA OR "Fit fOR The Aged" OR PRISCUS OR NORGEP):ti,ab,kw AND ("Emergency Medicine" OR "Emergency Department*" OR "trauma cent*" OR emergicenter* OR triag* OR "ED" OR "ER" OR "emergency" OR "emergencies" OR "A&E" OR "pre-hospital" OR prehospital OR EMS OR EMT OR ambulance* OR ((emergency OR emergencies OR ems OR ed OR er) AND (service* OR dispatch* OR department* OR unit* OR ward* OR room* OR center* OR centre* OR system* OR visit* OR admiss* OR admit* OR consult*)) OR (trauma AND (support OR center OR centre OR department* OR unit* OR room*))):ti,ab,kw AND (geriatr* OR geront* OR elder* OR old OR older OR oldest OR eldest OR senior* OR aged* OR aging OR agism OR "age factor*" OR "age-stratified" OR "age-related" OR "age-associated" OR "aging-related" OR retired OR retirement OR Retiree* OR "social security" OR "nursing home*" OR "assisted living" OR pension* OR senium* OR senil* OR dementia OR grandparent* OR grandmother* OR grandfather* OR grandma* OR grandpa* OR sexagenarian* OR septuagenarian* OR octagenarian* OR nonagenarian* OR centenarian* OR supercentenarian*):ti,ab,kw

© 2025 Skains RM et al. *JAMA Network Open*.

eFigure 1. Funnel Plots and Fail-Safe N Calculation Using Rosenberg Approach of Meta-Analyses

A. Clinical Pharmacist Review – Mean in Hospital in Length of Stay Days

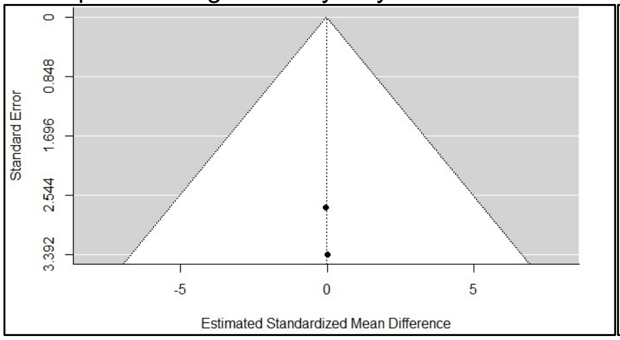

B. Clinical Pharmacist Review – Hospital Difference Admission Rates

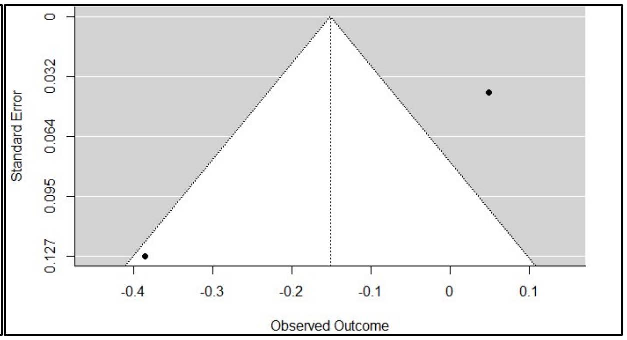

C. Clinical Pharmacist Review – PIM Deprescribing Rates

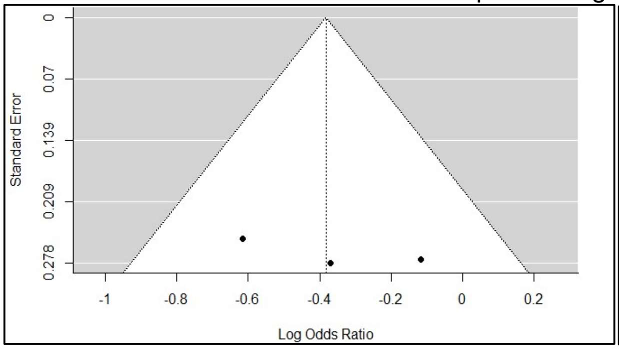

The Fail-Safe N analysis showed a significant effect for clinical pharmacist review on PIM deprescribing (average effect size: -.383, P=.013). The Fail-Safe N for the intervention effect estimates indicated that 2 negative unpublished studies would need to be added to change meta-analysis results to be nonsignificant, providing reassurance of the validity of our results.

D. Clinician Educational Intervention – PIM Prescribing Rates

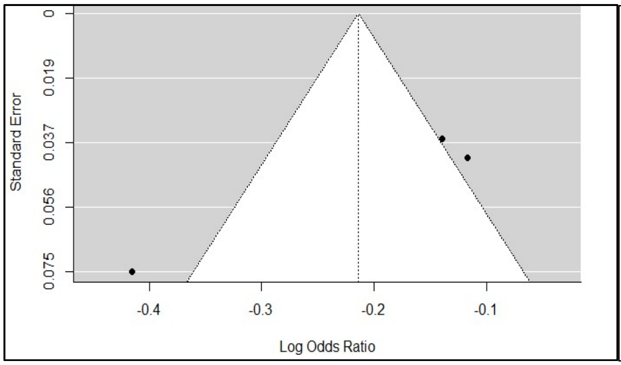

The Fail-Safe N analysis showed a significant effect for clinician educational interventions on PIM prescribing (average effect size: -.163, P<.0001). The Fail-Safe N for the intervention effect estimates indicated that 29 negative unpublished studies would need to be added to change meta-analysis results to be nonsignificant, providing reassurance of the validity of our results.

E. Computerized CDSS – PIM Ordering Rates

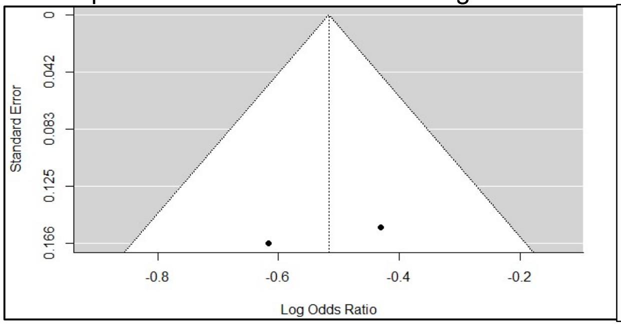

The Fail-Safe N analysis showed a significant effect for computerized CDSS on PIM ordering (average effect size: -.517, P<.0001). The Fail-Safe N for the intervention effect estimates indicated that 9 negative unpublished studies would need to be added to change meta-analysis results to be nonsignificant, providing reassurance of the validity of our results.

© 2025 Skains RM et al. *JAMA Network Open*.

F. FRID Review – Fall Recurrence at 12 Mos

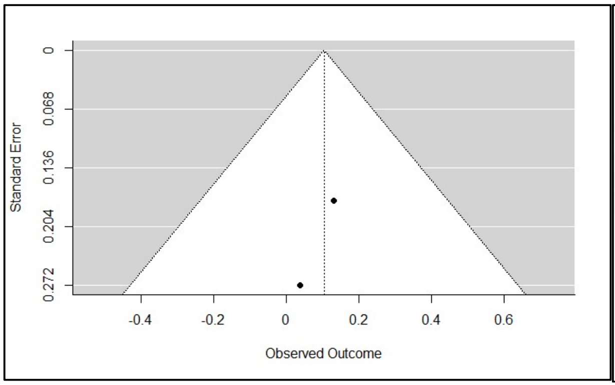

G. FRID Review – Time to First Fall

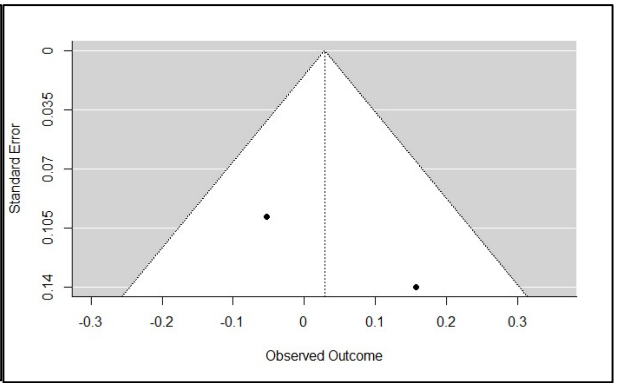

Legend:

Abbreviations: CDSS, clinical decision support systems; FRID, fall-risk increasing drug; Mos, months; PIM, potentially inappropriate medications

Figures show funnel plots and Fail-Safe N (calculation using Rosenberg approach) to assess presence of study bias and robustness of results in meta-analyses.

© 2025 Skains RM et al. *JAMA Network Open*.

eFigure 2. Results of Random-Effects Meta-Analysis Models

A. Clinical Pharmacist Review – Mean Difference in Hospital in Length of Stay Days

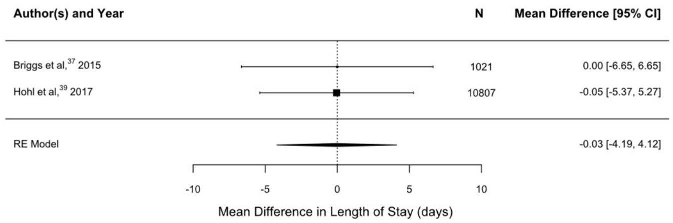

B. Clinical Pharmacist Review – Hospital Admission Rates

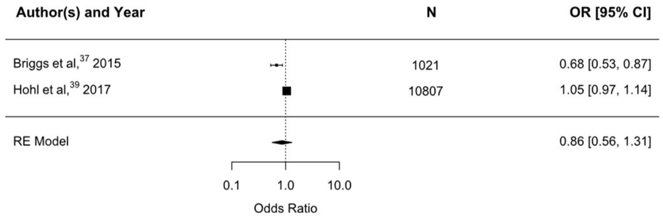

C. FRID Review – Fall Recurrence at 12 Mos

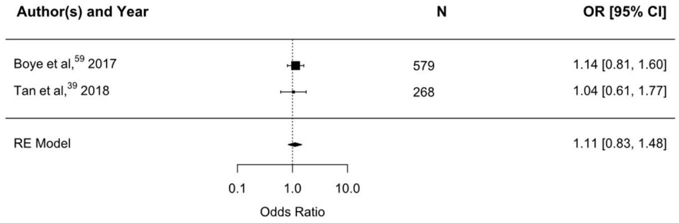

D. FRID Review – Time to First Fall

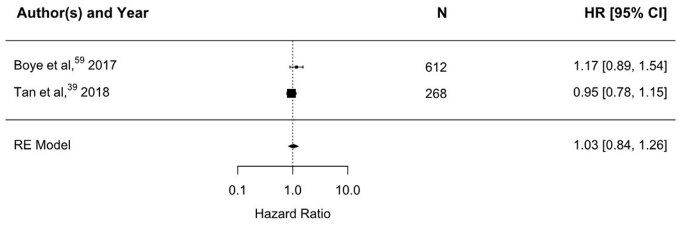

Legend:

Abbreviations: FRID, fall-risk increasing drug; HR, hazard ratio; Mos, months

© 2025 Skains RM et al. *JAMA Network Open*.

eFigure 3. Extracted Data from Source

Study details		Free text for additional info			Intervention	Comparator	Reported outcomes and treatment effect [include 95% CI]	Other Secondary Outcomes
Country								
Year of start								
Year of end								
Prospective/retrospective								
Study design								
**Population included in our analysis**								
Age (mean/median)					Intervention	Control	Total	
Number included in analysis				Predictor			0	
% male				Without predictor			#VALUE!	
Inclusion Criteria				Total		0		
Exclusion Criteria								
				Predictor			0	
				No predictor			#VALUE!	
**Predictors**				Total		0		
# patients with								
# pts w/				Predictor			0	
# pts w/				No predictor			#VALUE!	
# pts w/				Total		0		
# pts w/		fill as needed						
# pts w/		fill as needed		Predictor			0	
# pts w/		fill as needed		No predictor			#VALUE!	
# pts w/		fill as needed		Total		0		

© 2025 Skains RM et al. *JAMA Network Open*.

eTable. Included Studies Inclusion and Exclusion Criteria

Source	Inclusion criteria	Exclusion criteria
**Clinical pharmacist review studies**		
Atey et al,[45] 2023 Australia	Patients ≥65 y who presented to ED with subsequent admission to an acute medical unit (general medicine, EM, or mental health), were taking ≥1 regular medication prior to hospitalization, and received their first medication reconciliation on the ward within 48 h after transferring from ED	Patients: 1) not admitted to hospital from the ED, 2) admitted to units other than an eligible unit, 3) had overnight ED presentation between 9pm and 8am (ie, outside pharmacist working hours), 4) did not receive MedRec within 48hr after ED transfer, or 5) had incomplete data, eg, incomplete discharge summary
Briggs et al,[37] 2015 Australia	Patients ≥70 y, lived at home, reported ≥5 medications daily	Lived in residential aged care facility
Clementz et al,[43] 2019 France	Patients ≥75 y who were admitted to the OAEM unit for a fall, had >2 chronic diseases, and were taking ≥2 medications	Patients who required immediate resuscitation or palliative care
Hohl et al,[39] 2017 Canada	Consecutive high-risk patients ≥19 y presenting when a medication review pharmacist was on duty	Patients requiring immediate resuscitation according to the CTAS, multisystem trauma, a scheduled re-visit, sexual assault, postsurgical or pregnancy-related complication, social problems, or unable to link to administrative records
Jovevski et al,[42] 2023 United States (IN)	Preintervention group: patients ≥75 y who visited the ED between 10/1/2019 and 10/1/2020; had a positive ISAR score (≥3); and were discharged home<br>Postintervention group: patients ≥75 y who visited the ED between 2/12021 and 2/12022; had a positive ISAR score; were discharged home; and had a pharmacy medication review performed and documented in the EHR	Receiving hospice, palliative care, admitted from ED or did not have medication list in EHR
Kitchen et al,[40] 2020 Canada	All patients categorized as high-risk of presenting with an ADE according to a validated decision rule, ≥19 y, and presented to the ED when a clinical pharmacist was on shift	Patients with CTAS of 1 (ie, resuscitation), multisystem trauma, scheduled visits, sexual assaults, postsurgical or pregnancy-related complications, social problems, and duplicate visits; patients who died on arrival, left against medical advice, or lived out-of-province; any months of individuals' data following their death during the 12-mo follow-up
Marks et al,[38] 2021 United States	Patients ≥65 y who presented to the ED within 7 d of fall and were likely to be discharged from ED	Altered mental status; undomiciled; could not provide phone number for follow-up
Santolaya-Perrín et al,[41] 2019 Spain	Patients ≥65 y presenting to the ED of the participating sites, seen in the observation unit, and taking ≥1 outpatient drug for a condition that had lasted ≥6 mo	Patients managed by the Psychiatry Unit, in follow-up for palliative care, inability to communicate due to language despite interpreter or cognitive decline, follow-up of chronic conditions conducted in a private medical center, patients who did not sign informed consent, no written source of information available about the treatment
Shaw et al,[44] 2016 United States (CO)	6 mo of continuous Kaiser Permanente Colorado membership, ≥65 y, treated in hospital	Trauma, acute mycardial infarction, stroke, intoxication
**Geriatrician teleconsultation study**		
Matz et al,[46] 2021 Germany	Patients ≥70 y admitted to interdisciplinary<br>ED of a single tertiary care medical center with an ISAR≥2	No written declaration of consent; participation in other clinical trials; dependency or employment relationship with the sponsor; forced admission due to a psychiatric illness
**Geriatrician teleconsultation study**		
Biese et al,[47] 2011 United States (NC)	Patients ≥65 y presenting to ED of primary training facility in May 2010 who underwent chemical sedation and Foley catheter placement	NA

© 2025 Skains RM et al. *JAMA Network Open*.

Goldberg et al,[48] 2022 United States (RI)	Clinicians who prescribed medications to ED patients ≥65 y during the pre-and post-intervention periods	NA
Moss et al,[49] 2019 United States (NC)	All ED discharge medications to patients ≥65 y discharged from the ED prescribed by both the trained and untrained residents	Medications administered in the ED were not included
O'Connor et al,[51] 2021 United Kingdom	Patients ≥65 y admitted to the CDU, an ED	NA
Stevens et al,[5] 2017 United States (Multisite)	Patients ≥65 y treated and discharged from ED	NA
Vandenberg et al,[52] 2024 United States (multisite)	Patients ≥65 y discharged from the ED	NA
Vaughan et al,[50] 2021 United States (GA)	Patients ≥65 y discharged from the ED	NA
Vaughan et al,[53] 2023 United States (multisite)	Patients ≥65 y discharged from the ED	NA
**Computerized decision support system studies**		
Griffey et al,[55] 2012 United States (MA)	Patients ≥65 y who had order for a medication in the knowledge base during the study period	Patient orders in which qualifying medication orders were subsequently cancelled and any orders with missing data
Kim et al,[56] 2017 United States (WA)	Patients ≥65 y	NA
Liu et al,[57] 2019 Taiwan	Patients ≥65 y awaiting hospitalization after ED diagnosis and treatment; patients with polypharmacy (≥10 medications) or PIMs	Not described
Terrell et al,[58] 2009 United States (IN)	EM faculty and resident physicians Patient population: ≥65 y prescribed a targeted PIM at ED discharge	3 EM faculty physicians who participated in the conduct of the study were excluded and all PGY-1 resident physicians
**FRID review studies**		
Boyé et al,[59] 2017 the Netherlands	Patients ≥65 y, community-dwelling, ED-visit because of a fall, and use of ≥1 FRID	Participation in another trial; fall not meeting criteria of specified definition; anticipated problems with follow-up; not willing to complete research protocol
Polinder et al,[60] 2016 the Netherlands	Patients ≥65 y, visited the ED due to a fall, use of ≥1 FRID, mini-mental state examination score of ≥21/30 points, ability to walk independently, community dwelling, written informed consent	Participation in another trial; fall not meeting criteria of specified definition; anticipated problems with follow-up; not willing to complete research protocol
Tan et al,[61] 2018 Malaysia	Community-dwelling ED patients ≥65 y with ≥2 falls or 1 injurious fall in the past 12 mo	Clinically-diagnosed dementia, major psychiatric illnesses, and/or inability to stand

© 2025 Skains RM et al. *JAMA Network Open*.
