# Supplementary material for: Emergency Department Programs to Support Medication Safety in Older Adults: A Systematic Review and Meta-Analysis
Source: JAMA Netw Open. 2025 Mar 11;8(3):e250814. doi: 10.1001/jamanetworkopen.2025.0814 (PMC11897843; doi:10.1001/jamanetworkopen.2025.0814)
Supplement: Supplement 2. — Nonauthor Collaborators [file jamanetwopen-e250814-s002.pdf]

Supplemental Online Content: Nonauthor Collaborators
*First name, last name, and suffix (if applicable) are required and will appear in PubMed.

*Group Name(s): Geriatric Emergency Department Medication Safety Guidelines Group							
***First Name and Middle Initial(s)**	***Last Name**	***Suffix (eg, Jr, III)**	Academic Degrees	Institution	Location (city, state/province, country)	Role or Contribution, eg, chair, principal investigator	Group (if more than 1 Group listed in the byline) and/or Subgroup (eg, Steering Committee)
Zachary	Cardon		MD	Department of Emergency Medicine, University of North Carolina School of Medicine	Chapel Hill, North Carolina, USA	Concept and design	Geriatric Emergency Department Guidelines Medication Safety Group
Jon B.	Cole		MD	Department of Emergency Medicine, Hennepin Healthcare and University of Minnesota Medical School	Minneapolis, Minnesota, USA	Concept and design	Geriatric Emergency Department Guidelines Medication Safety Group
Ming	Ding		MBBS, DSc	Department of Emergency Medicine, University of North Carolina School of Medicine	Chapel Hill, North Carolina, USA	Concept and design	Geriatric Emergency Department Guidelines Medication Safety Group
Natalie M.	Elder		MD	Department of Emergency Medicine, The Larner College of Medicine at the University of Vermont	Burlington, Vermont, USA	Concept and design	Geriatric Emergency Department Guidelines Medication Safety Group
Alexander	Fenn		MD, MA	Department of Emergency Medicine, University of North Carolina School of Medicine	Chapel Hill, North Carolina, USA	Concept and design	Geriatric Emergency Department Guidelines Medication Safety Group
Naira	Goukasian		MD	Department of Emergency Medicine, University of North Carolina School of Medicine	Chapel Hill, North Carolina, USA	Concept and design	Geriatric Emergency Department Guidelines Medication Safety Group
Danya	Khoujah		MBBS, MEHP	Department of Emergency Medicine, University of Maryland School of Medicine	Baltimore, Maryland, USA	Concept and design	Geriatric Emergency Department Guidelines Medication Safety Group

Supplemental Online Content: Nonauthor Collaborators
*First name, last name, and suffix (if applicable) are required and will appear in PubMed.

***First Name and Middle Initial(s)**	***Last Name**	***Suffix (eg, Jr, III)**	Academic Degrees	Institution	Location (city, state/province, country)	Role or Contribution, eg, chair, principal investigator	Group (if more than 1 Group listed in the byline) and/or Subgroup (eg, Steering Committee)
Jennifer L.	Koehl		PharmD, BCPS	Department of Emergency Medicine, Massachusetts General Hospital, Harvard Medical School	Boston, Massachusetts, USA	Concept and design	Geriatric Emergency Department Guidelines Medication Safety Group
Joshua	Niznik		PharmD, PhD	Division of Pharmaceutical Outcomes and Policy, University of North Carolina at Chapel Hill, Eshelman School of Pharmacy	Chapel Hill, North Carolina, USA	Concept and design	Geriatric Emergency Department Guidelines Medication Safety Group
Katren	Tyler		MD	Department of Emergency Medicine, University of California Davis School of Medicine	Sacramento, California, USA	Concept and design	Geriatric Emergency Department Guidelines Medication Safety Group
