# Supplementary material for: Emergency Department Programs to Support Medication Safety in Older Adults: A Systematic Review and Meta-Analysis
Source: JAMA Netw Open. 2025 Mar 11;8(3):e250814. doi: 10.1001/jamanetworkopen.2025.0814 (PMC11897843; doi:10.1001/jamanetworkopen.2025.0814)
Supplement: Supplement 3. — Data Sharing Statement [file jamanetwopen-e250814-s003.pdf]

Data Sharing Statement

Skains. Emergency Department Programs to Support Medication Safety in Older Adults. *JAMA Netw Open*. Published March 11, 2025. doi:10.1001/jamanetworkopen.2025.0814

Data
Data available: Yes
Data types: Data (not involving human participants)
How to access data: rskains@uabmc.edu
When available: With publication

Supporting Documents
Document types: None

Additional Information
Who can access the data: Researchers whose proposed use of the data has been approved.
Types of analyses: For research purposes.
Mechanisms of data availability: With investigator support and approval of proposal.
Any additional restrictions: None.
